# FGF23 is synthesised locally by renal tubules and activates injury-primed fibroblasts

**DOI:** 10.1038/s41598-017-02709-w

**Published:** 2017-06-13

**Authors:** Edward R. Smith, Sven-Jean Tan, Stephen G. Holt, Tim D. Hewitson

**Affiliations:** 1 Department of Nephrology, The Royal Melbourne Hospital, Melbourne, Victoria Australia; 2 Department of Medicine - Royal Melbourne Hospital, University of Melbourne, Melbourne, Victoria Australia

## Abstract

In kidney disease, higher circulating levels of the mineral-regulating hormone fibroblast growth factor (FGF)-23 are predictive of disease progression but direct pathogenic effects on the kidney are unknown. We sought evidence of local renal synthesis in response to unilateral ureteric obstruction in the mouse, and pro-fibrotic actions of FGF23 on the fibroblast *in vitro*. Acute tubulointerstitial injury due to unilateral ureteric obstruction stimulated renal FGF23 synthesis by tubules, and downregulated inactivating proprotein convertases, without effects on systemic mineral metabolism. *In vitro*, FGF23 had divergent effects on fibroblast activation in cells derived from normal and obstructed kidneys. While FGF23 failed to stimulate fibrogenesis in normal fibroblasts, in those primed by injury, FGF23 induced pro-fibrotic signalling cascades via activation of TGF-β pathways. Effects were independent of α-klotho. Tubule-derived FGF23 may amplify myofibroblast activation in acute renal injury, and might provide a novel therapeutic target in renal fibrosis.

## Introduction

Fibroblast growth factor 23 (FGF23) is a bone-derived member of the endocrine FGF family known to regulate mineral metabolism principally through effects in the kidney and parathyroid glands^1^. Physiologically, FGF23 signals through interactions with one of several FGF receptors (FGFR), in complex with α-klotho (αKL), a co-receptor that confers tissue specificity^2^. The hormone circulates as both an intact molecule and carboxy-terminal fragments^3^, generated through cleavage at R^176^XXR^179^ by proprotein convertases^4^.

FGF23 levels rise early in Chronic Kidney Disease (CKD), predominantly as intact protein^3^, and generally precede changes in other mineral metabolites^5^. Higher levels are associated with cardiovascular(CV) and non-CV mortality^6, 7^, as well as progression to end-stage kidney disease^8–11^, all independent of other mineral and renal confounders. Similarly, circulating FGF23 concentrations rise rapidly in acute kidney injury^12^, again preceding changes in other mineral markers and contemporary measures of renal function^13^. The kidney appears to play a central role in FGF23 metabolism, and appears to be the only site of circulating hormone removal in humans. FGF23 levels therefore seem inextricably linked to renal function.

Although epidemiological data correlating FGF23 with poor outcomes is unequivocal, the aetiology of the initial rise in FGF23 in early kidney disease remains unclear^14^. While uraemia clearly enhances FGF23 expression in bone^15^, circulating levels appear to rise before increments in osteocytic FGF23 expression in some animal models of spontaneous progressive CKD^16^. Thus, the cause of intact FGF23 excess seen in CKD is complex and likely to include increased osteocytic production in response to local changes in mineralisation^17, 18^, inflammation and functional iron deficiency via hypoxia inducible factor (HIF)1α activation^19^, and altered handling by the kidney^20^. Whether elevated FGF23 concentrations serve as a surrogate for derangements in mineral metabolism that are associated with outcome, or are directly involved in the pathogenesis of cardiovascular disease (CVD) or CKD is widely debated but unproven. With respect to CVD, the available evidence is consistent with the notion that FGF23 increases in CKD, initially as an adaptive response to maintain mineral homeostasis, but eventually becomes maladaptive driving collateral damage in the heart^21^. Here, recent landmark studies suggest that, independent of αKL, FGF23 excess can drive off-target hypertrophic gene programmes in the cardiomyocyte, which result in left ventricular hypertrophy, fibrosis and dysfunction^22^.

That initial increases in circulating FGF23 concentrations are not completely explained by increased bone synthesis has also led to a search for possible extra-osseous sources. Renal expression of FGF23 is contentious, with some reports demonstrating very low-level physiological expression^23–25^, but this is not a universal finding^26–29^. Furthermore, while renal FGF23 expression has been noted in several models of CKD^28, 29^, the direct relationship, if any, between FGF23 and renal pathology is unknown. Comparative transcriptome analysis of kidney tissue from CKD and non-CKD models of FGF23 excess have identified the activation of common pathways associated with fibrosis and inflammation, including transforming growth factor (TGF)-β1 related signalling^30^, a master regulator of fibrosis^31^.

To investigate the putative role of FGF23 as a mediator of renal fibrosis, we sought evidence of ectopic renal production of FGF23 in response to unilateral ureteric obstruction (UUO), and determined the effects of FGF23 on renal interstitial fibroblast activation and function *in vitro*.

## Results

### UUO in mice causes renal fibrogenesis without changes in systemic mineral handling

**Figure 1:**
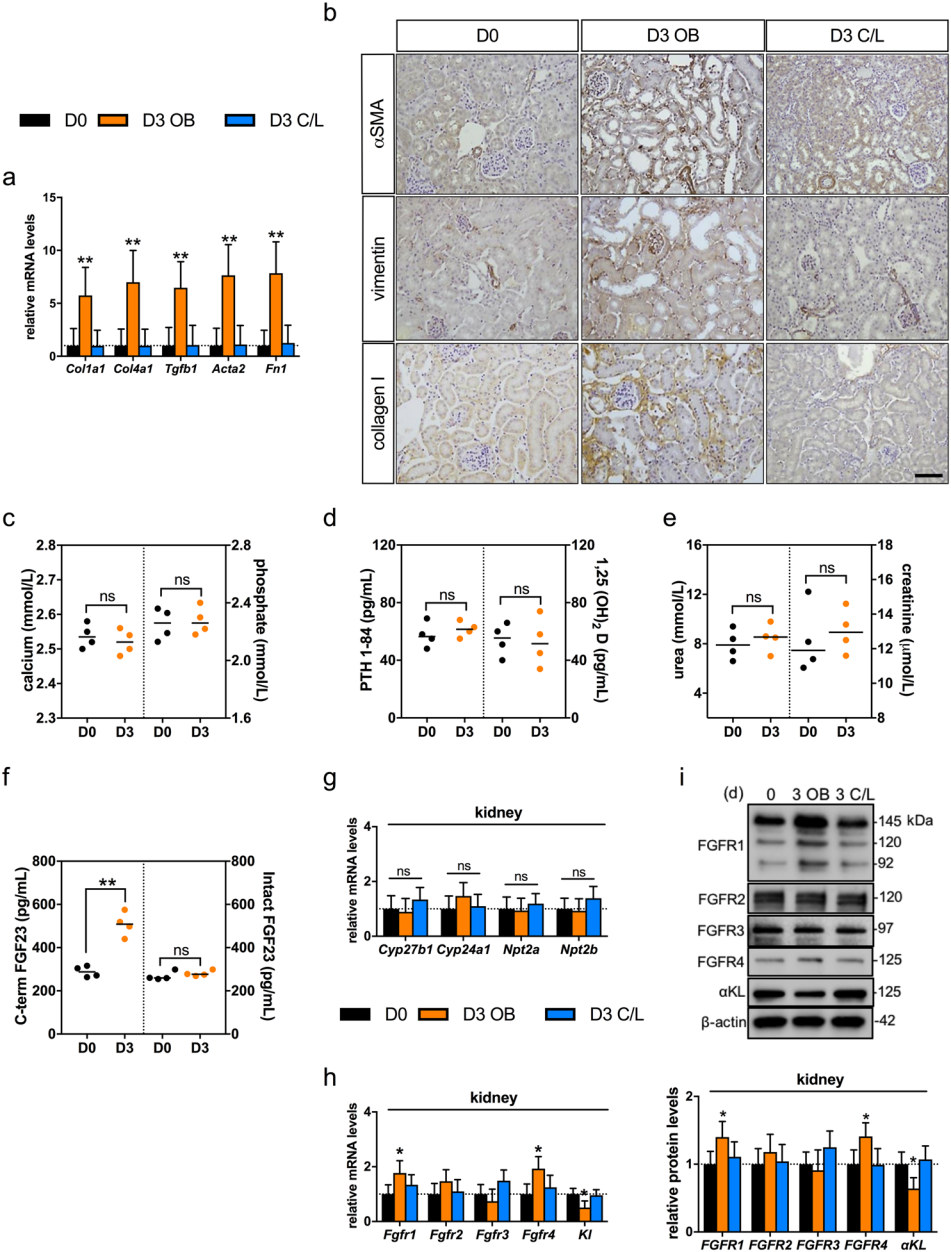
UUO has no acute effect on systemic mineral metabolism in mice. (**a**) qRT-PCR analysis of fibrosis-related gene expression in tissue isolated 3 days post UUO (D3) from obstructed (OB) and unobstructed contralateral (C/L) kidneys relative to levels in control animals (D0). Fibrogenic targets were collagen alpha-1(I) chain (*Col1a1*), collagen alpha-1(IV) chain (*Col4a1*), transforming growth factor-β_1_ (*Tgf b1*), α-smooth muscle actin (*Acta2*) and fibronectin (*Fn1*) (n = 4 mice for each group). (**b**) Representative DAB immunohistochemical staining for αSMA, vimentin and collagen I in control D0, D3 OB and D3 C/L kidneys. Counter staining was performed with haemotoxylin. Scale bar: 100μm. (**c–f**) Serum levels of calcium and phosphate (**c**), PTH 1–84 and 1,25(OH)_2_ vitamin D (**d**), urea and creatinine (**e**) and intact and C-terminal FGF23 (**f**) in control D0 animals and D3 UUO animals (n = 4 mice for each group). (**g**,**h**) qRT-PCR analysis of FGF23 target gene expression (**g**) and FGFR receptor/co-receptor gene expression (**h**) in tissue from obstructed (D3 OB) and unobstructed contralateral kidneys (D3 C/L) relative to transcript levels in control animals (D0). FGF23 target gene expression transcripts included 25-hydroxyvitamin D-1 alpha hydroxylase (*Cyp27b1*), 1,25-dihydroxyvitamin D(3) 24-hydroxylase (*Cyp24a1*), Sodium-dependent phosphate transport protein 2A (*Npt2a*) and Sodium-dependent phosphate transport protein 2B (*Npt2b*). Fibroblast growth factor receptor (FGFR)/co-receptor targets included *Fgfr1-4* and *a*-Klotho (Kl). (n = 4 mice for each group). (**i**) Representative Western blot analysis of FGFR receptor/co-receptor levels in D0, D3 OB and D3 C/L kidney homogenates. β-tubulin was used as a loading control. Quantification of the receptor/β-tubulin signal ratios normalised (=1.0) to control D0 levels (n = 4 mice for each group). Data are shown as mean ± SD. *P < 0.05; **P < 0.01; ns, not significant. P-values were determined in (**a**,**g**–**i**) by one-way ANOVA with Holm-Sidak multiple comparisons test; (**c**–**f**), by two-tailed unpaired Student’s *t*-test.

Acute injury resulted in a ~3–8 fold increase in the expression of fibrogenic markers in the obstructed kidney (Fig. 1a; n = 4 each group), with expression unchanged in the contralateral (C/L) unobstructed kidney (Fig. 1a).

*De novo* expression of αSMA in fibroblasts indicates myofibroblast differentiation^32^, a hallmark of fibroblast activation. In accordance with the well-described natural history of the model^33^, an acute recruitment of myofibroblasts was seen with *de novo* interstitial staining for αSMA and vimentin, a more generic marker of fibroblasts, at day 3 (D3) post-UUO. Neither was found in the interstitium of the C/L unobstructed kidney, nor the normal (D0) kidney, confirming an absence of fibroblasts. Recruitment of myofibroblasts was paralleled by an increase in interstitial collagen I staining, mirroring changes in gene expression (Fig. 1b).

Next, we investigated whether UUO had any effect on serum and tissue parameters of mineral handling. Three days of UUO had no effect on serum calcium, phosphate, PTH, and vitamin D (Fig. 1c,d). The only change in mineral-related parameters was an isolated increase in C-terminal FGF23, with no change in intact (bioactive) FGF23 (Fig. 1f). The presence of a C/L intact kidney maintained normal renal function (Fig. 1e). The mRNA expression of downstream targets of FGF23 signalling were all unaltered by UUO (Fig. 1g), although there was a modest increase in mRNA and protein levels of FGFR1 and 4 and a reduction in expression of the co-receptor αKL in the injured kidney (Fig. 1h,i).

### FGF23 is expressed in the obstructed kidney following UUO

**Figure 2:**
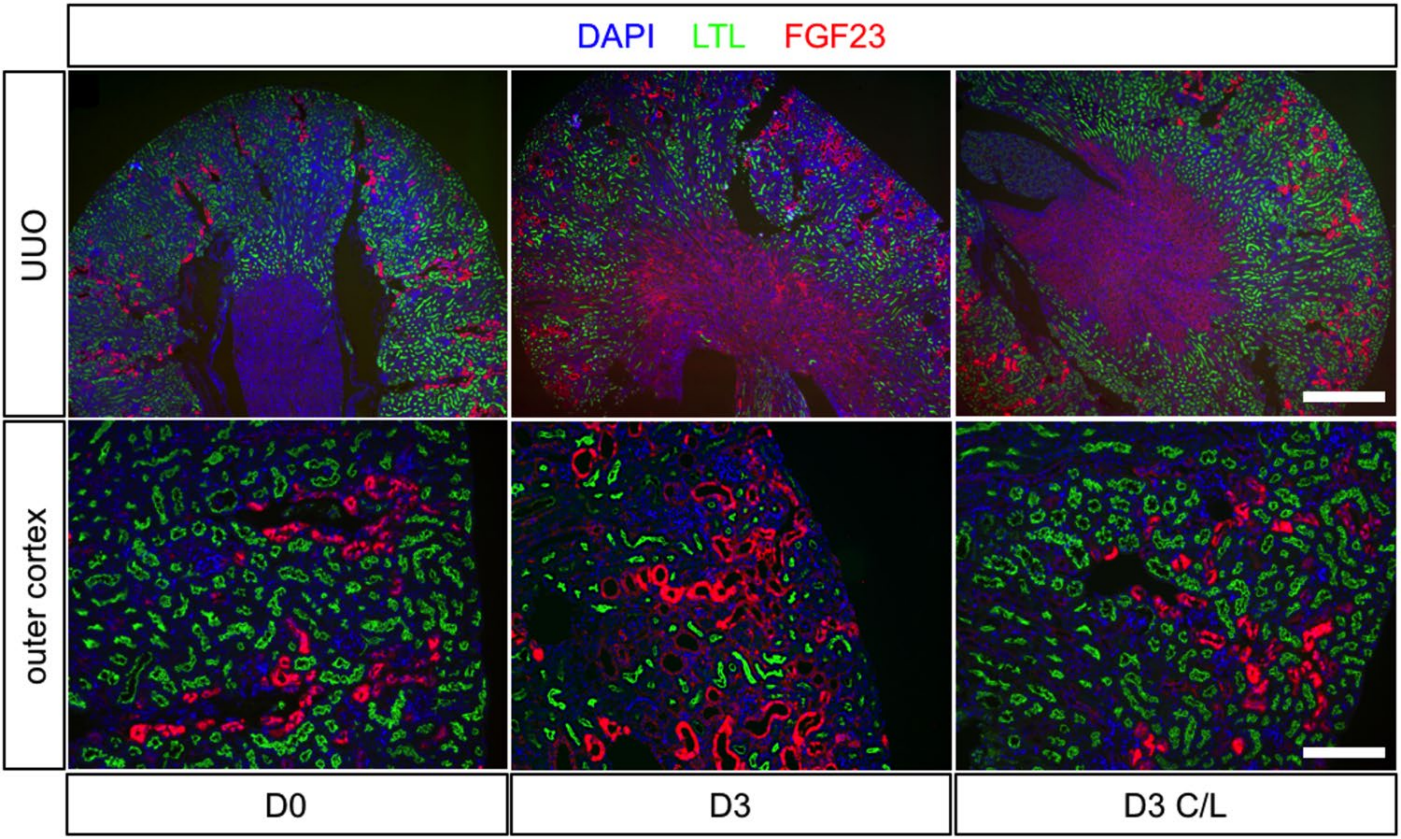
Renal localisation of FGF23 before and after UUO. Representative immunofluorescent staining of FGF23 (red), a proximal tubule marker LTL (green), and the nuclear marker DAPI (blue) in D0, D3 OB and D3 C/L kidney sections. Lower panel shows enlarged region of the outer cortex from corresponding micrographs above. Scale bar upper panel = 250 μm; lower panel = 100 μm.

Acute changes in staining for FGF23 were identified following UUO (Fig. 2). At D0, staining was predominantly localised to the apical surface of tubules of the outer cortex. Cortical staining increased in the obstructed and unobstructed C/L kidney at D3, with a parallel increase in basolateral staining in the medulla (see Supplementary Fig. S1). There was no staining in glomeruli before or after UUO. Co-staining with the lectin lotus tetragonolobus (LTL), a specific marker of brush borders in proximal tubules, showed that FGF23 staining was not localised to these segments. There was, however, a decrease in cortical staining for LTL in the obstructed kidney after UUO, while the staining in the C/L kidney remained similar to that in the D0 animals. We are unable to say if this lack of co-localisation after UUO was simply due to a loss of brush border in proximal convoluted tubules (denuded), or actual LTL negative nephron segments.

**Figure 3:**
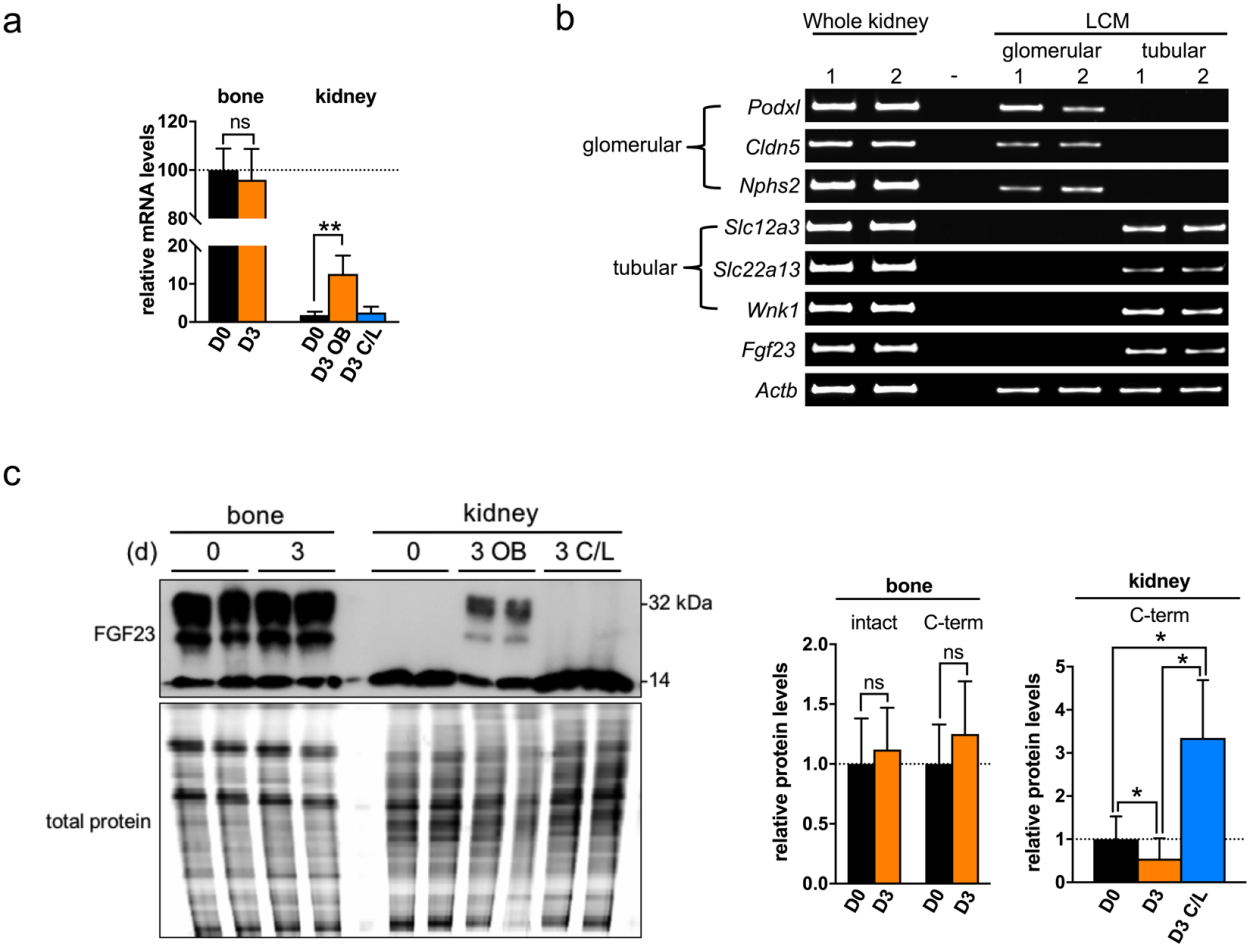
Acute tubulointerstitial injury induces *de novo* tubular FGF23 expression. (**a**) qRT-PCR analysis of FGF23 gene expression in both hind limb bone (D0, D3) and kidney tissue. Results are expressed relative to the bone transcript levels (=100) in D0 animals (n = 4 mice for each group). (**b**) RT-PCR detection of *Fgf23* and glomerular- and tubular-enriched transcripts in whole kidney homogenates and laser capture microdissected isolates from D3 OB tissue (n = 2 animals). Glomerular-enriched transcripts were podocalyxin-like protein 1 (*Podxl*), claudin 5 (*Cldn5*) and podocin (*Nphs2*). Tubular -enriched transcripts were solute carrier family 12 member 3 (*Slc12a3*), solute carrier family 22 member 3 (*Slc22a13*), and serine/threonine-protein kinase WNK1 (*Wnk1*). β-actin (*Actb*) was used a positive control. (**c**) Representative Western blot analysis of FGF23 protein expression in both hind limb bone (D0, D3) and kidney. Antibody detects both full-length intact FGF23 (32 kDa) and C-terminal FGF23 fragment (14 kDa). Total protein was used as a loading control. Densitometric quantitation of the intact and C-terminal FGF23 ratios normalised (=1.0) to control D0 levels in bone and C-terminal FGF23 levels in kidney extracts are shown alongside (n = 4 mice for each group). Quantitation of intact FGF23 levels in kidney was not performed due to the very low levels detected. Data are shown as mean ± SD. *P < 0.05; **P < 0.01; ns, not significant. P-values were determined in (**a**,**c**) by one-way ANOVA with Holm-Sidak multiple comparisons test.

Renal mRNA expression of FGF23 increased in the obstructed kidney after UUO, although this remained several magnitudes less than bone expression (Fig. 3a). UUO had no effect on bone mRNA expression (Fig. 3a). While immunohistochemical staining localised FGF23 protein to tubules, it did not tell us if this was due to local expression, or glomerular filtration and absorption. To distinguish, we used laser capture micro-dissection (LCMD) to examine FGF23 mRNA expression in a distinct population of tubules and glomeruli. RT-PCR analysis of genes known to be maximally enriched in glomeruli and tubules^34^ confirmed integrity of the RNA, the anatomical specificity of micro-dissected samples, and the lack of contamination from surrounding tissue. In agreement with the staining pattern, LCMD showed that tubules, and not glomeruli, were a source of renal FGF23 mRNA (Fig. 3b).

**Figure 4:**
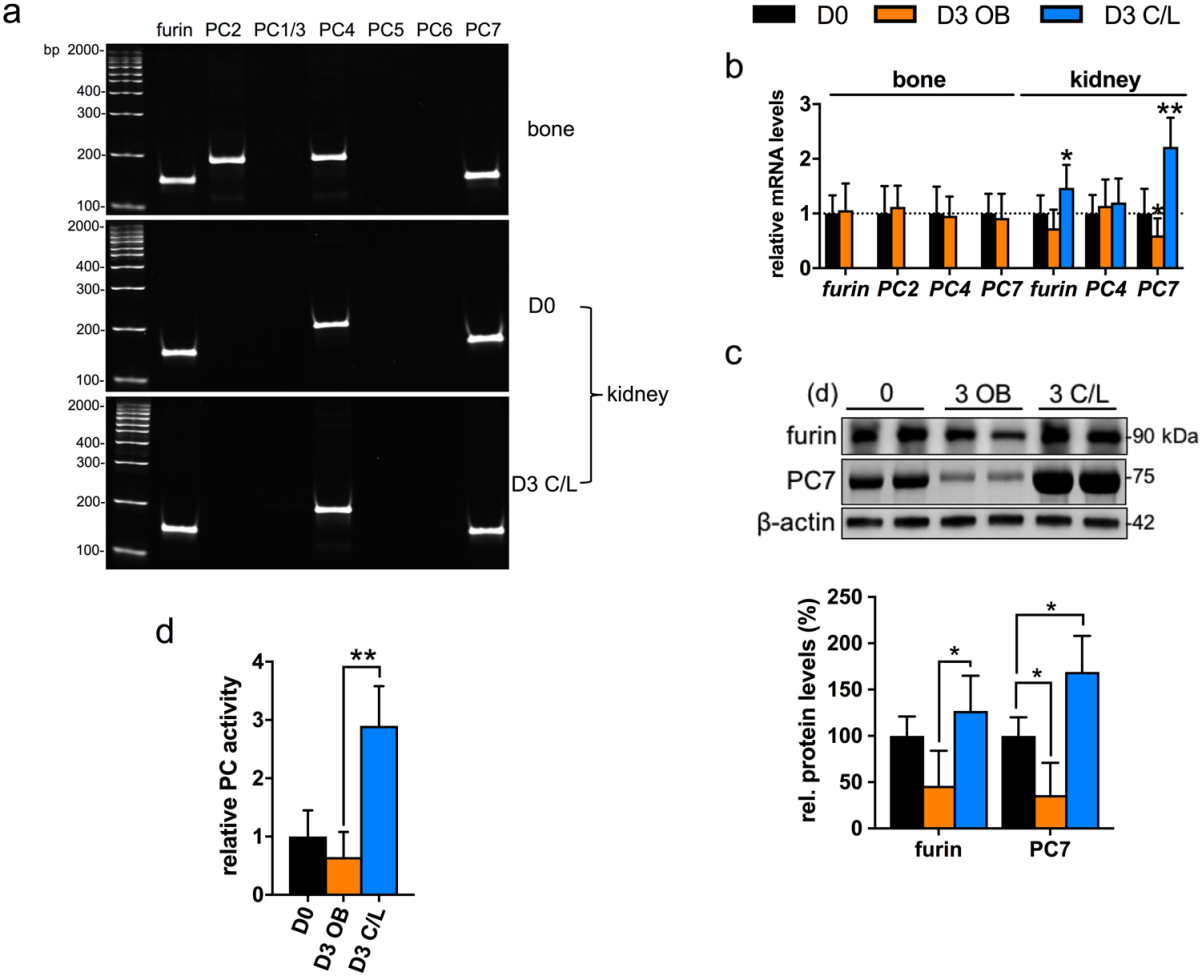
UUO induces changes in proprotein convertase expression. (**a**) RT-PCR detection of proprotein convertase (PC) transcripts expressed in mouse hind limb bone and kidney (D0 and D3 post-UUO). (**b**) qRT-PCR analysis of proprotein convertases (furin, PC2, PC4, PC7) gene expression in both hind limb bone (D0, D3) and kidney tissue at D0 and D3 (OB and C/L). Results are expressed relative to transcript levels at D0 (n = 4 mice for each group). (**c**) Representative Western blot analysis of furin and PC7 levels in kidney homogenates. β-tubulin was used as a loading control. Quantification of the proproprotein convertase/β-actin signal ratios normalised (=1.0) to control D0 levels (n = 4 mice for each group). (**d**) Relative proprotein convertase enzyme activity in D3 OB and D3 C/L kidney homogenates normalised to D0 controls (n = 4 mice for each group). Data are shown as mean ± SD. *P < 0.05; **P < 0.01. P-values were determined by one-way ANOVA with Holm-Sidak multiple comparisons test.

Western blotting of whole kidney lysates revealed that intact FGF23 (32 kDa) was virtually undetectable at D0, but clearly present in the obstructed kidney D3 post UUO (Fig. 3c). Conversely, levels of the C-terminal FGF23 fragment were increased in the unobstructed C/L kidney. This led us to examine the family of enzymes implicated in FGF23 cleavage (Fig. 4), the proprotein convertases (PC)^4, 35–37^, our focus being on those expressed in the kidney (Fig. 4a). We found a reduction in both mRNA (Fig. 4b) and protein levels (Fig. 4c) for furin and PC7 in the obstructed kidney, and a corresponding increase in expression of each in the unobstructed C/L kidney. Total enzyme activity, as measured by breakdown of a PC-specific fluorogenic substrate, was likewise increased in the C/L kidney (Fig. 4d).

### FGF23 induces (myo)fibroblast activation

**Figure 5:**
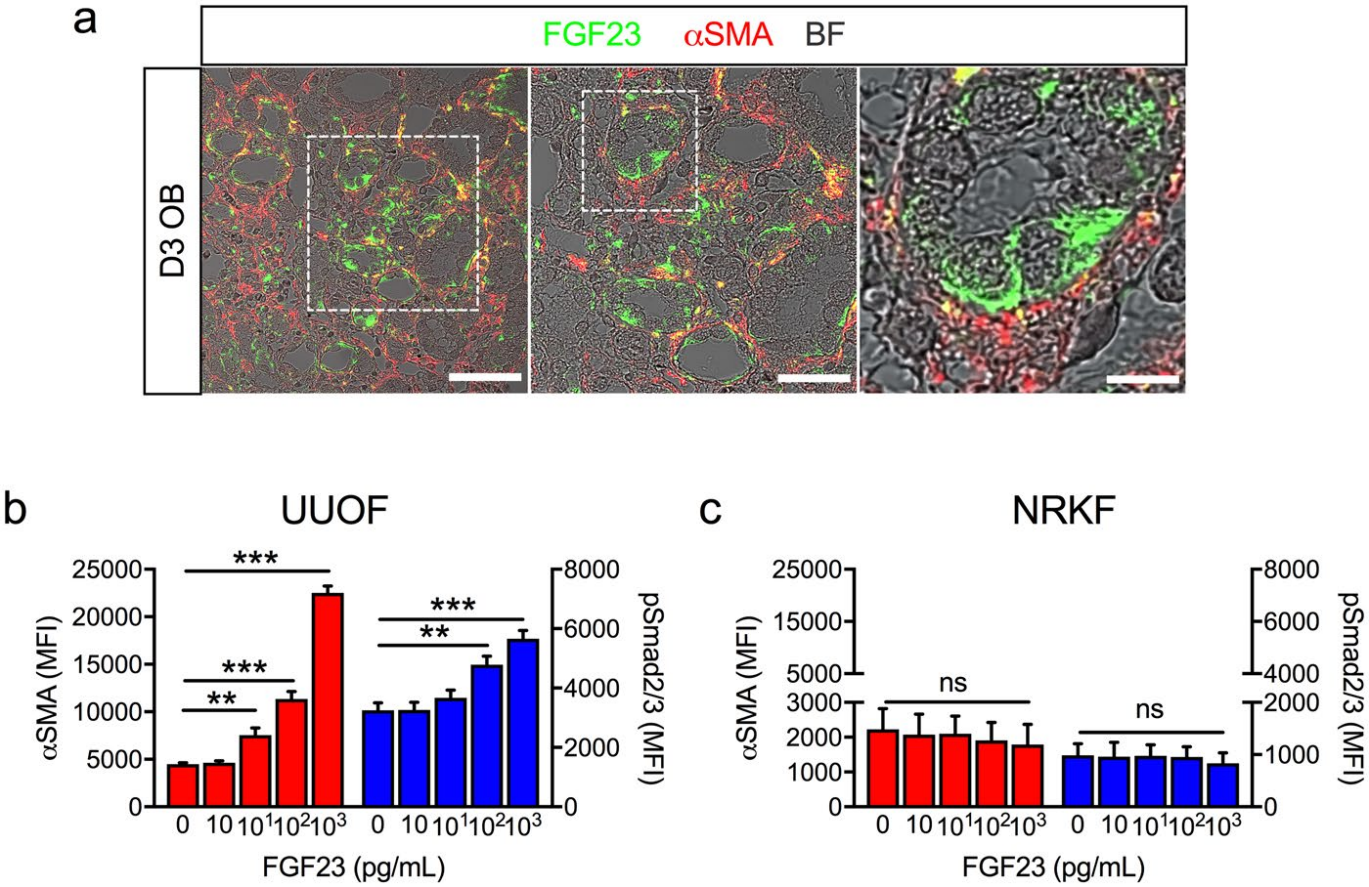
FGF23 stimulates renal fibroblast activation. (**a**) Representative immunofluorescent staining of FGF23 (green) and αSMA (red) in D3 OB kidney sections using brightfield (BF) confocal microscopy. Boxed region is enlarged in adjacent panels. Scale bars = 50, 25, 10 μm left to right. (**b**,**c**) Quantitative flow cytometric analysis of FGF23 dose-related changes in αSMA (red) and phosphorylated Smad2/3 (blue) staining (mean fluorescence intensity, MFI) in UUOF (**b**) and NRKF (**c**) after 48 h treatment (n = 4 independent experiments). Data are shown as mean ± SD and represent at least three independent experiments with similar results. **P < 0.01; ***P < 0.001; ns, not significant. P-values were determined in (**b,c**) by one-way ANOVA with Holm-Sidak multiple comparisons test.

Confocal imaging of co-staining for FGF23 and αSMA showed that tubule FGF23 was juxtapositioned with interstitial myofibroblasts (Fig. 5a), implying a potential interaction. Although FGF23 staining was primarily localised to the distal tubular epithelium (Fig. 2), at higher magnification cytoplasmic staining was also evident in some cells positive for αSMA, suggesting that myofibroblasts might also be a minor source of renal FGF23. These findings prompted us to examine the effects of FGF23 on fibroblast activation. Both exogenous TGF-β1 and FGF23 increased αSMA staining in rat fibroblasts derived from kidneys 3 days after UUO (UUOF) (see Supplementary Fig. S2). αSMA was organised into stress fibres, a defining characteristic of myofibroblasts. Intriguingly, the effects of FGF23 in UUOF were not apparent in fibroblasts isolated from normal kidneys (NRKF). In this case, while TGF-β1 caused a similar qualitative effect on αSMA expression, FGF23 had no effect (see Supplementary Fig. S2). Consistent with the cytochemistry, flow cytometry in UUOF showed that FGF23 produced a dose effect on αSMA and Smad2/3 phosphorylation, a downstream intermediate of canonical TGF-β1 signalling (Fig. 5b). Again, consistent with the staining pattern, FGF23 had no dose-related effect on αSMA or Smad2/3 phosphorylation in NRKF (Fig. 5c). FGF23 had no effect on the viability or proliferation of either cell type at the tested concentrations (see Supplementary Fig. S3).

### Differences in TGF-β1 signalling and receptor expression in fibroblasts isolated from normal and fibrotic kidneys

To examine the divergent effect of FGF23 on TGF-β1 signalling, we undertook qPCR profiling studies of common TGF-β1 signalling targets (see Supplementary Fig. S4). The pattern of gene expression in response to TGF-β1 was similar in NRKF and UUOF. However, while the response to FGF23 in UUOF resembled the response to TGF-β1 in these cells, the pattern in NRKF was quite different. There was an opposite effect in some instances, with αSMA (*Acta2*), TGF-β receptor II (*Tgfbr2)* and β-catenin *(Ctnnb1)* expression decreased more than 2–fold compared to the increase seen with TGF-β1, whereas the expression of *endoglin*, *myc* and *E2f5* showed a relative increase compared to suppression with TGF-β1 (see Supplementary Fig. S4). While FGF23 expression was undetectable in NRKF, low-transcript levels were detectable in UUOF basally, and augmented >25-fold in response to exogenous TGF-β1 (1 ng/mL), consistent with activated fibroblasts being a source of FGF23 *in vivo* (Fig. 5a). However, we were unable to confirm this at the protein level due to the lack of a validated anti-rat antibody.

**Figure 6:**
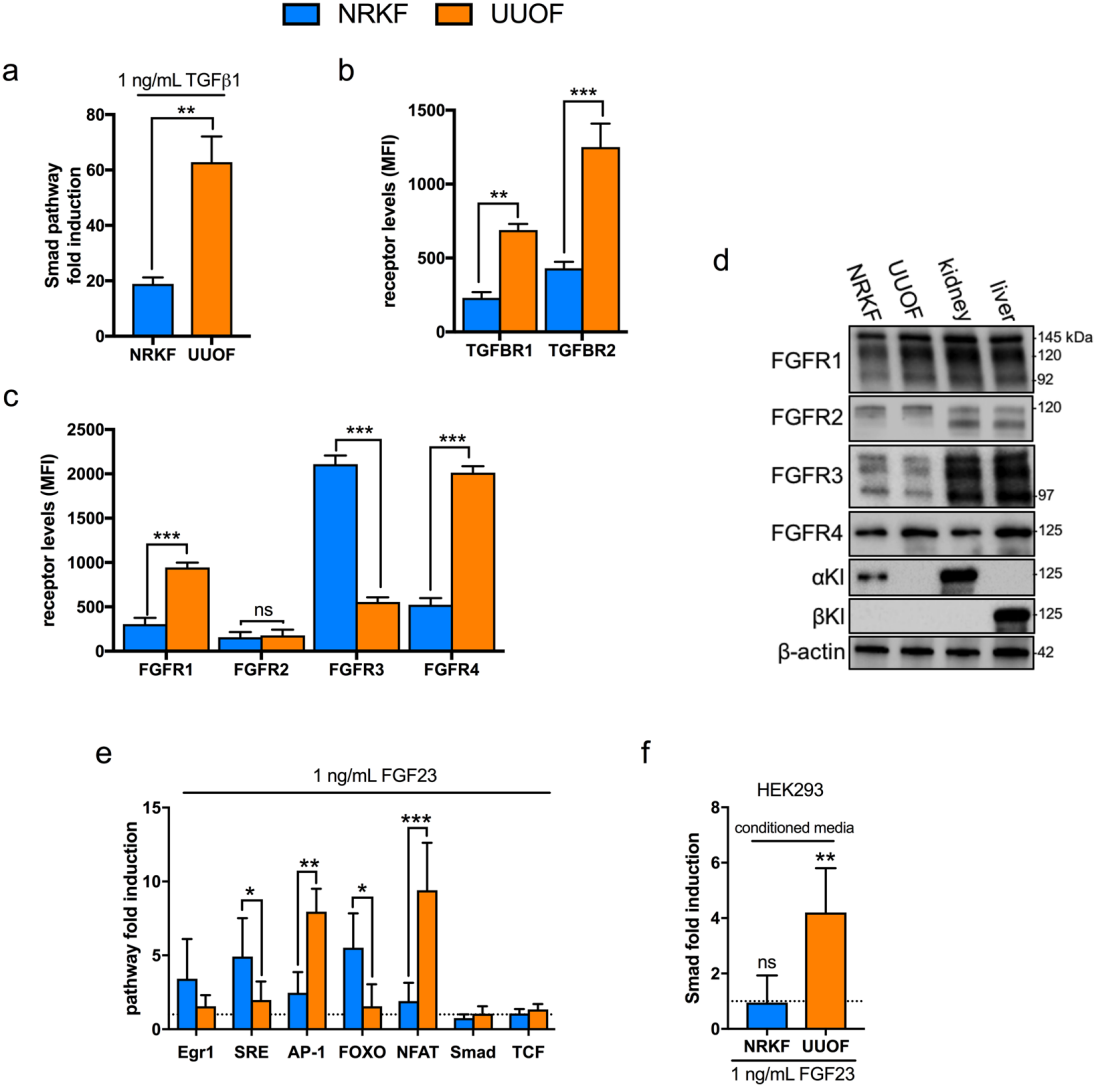
Differential expression of TGFβ and FGF receptors in NRKF and UUOF. **(a**) Fold change in TGF-β1 bioactivity in NRKF and UUOF transiently transfected with a Smad- responsive luciferase construct in response to treatment with exogenous TGF-β1 (1 ng/mL for 30 min). (**b**) Quantitative flow cytometric analysis of the cell-surface expression of TGFBR1 and TGFBR2 in NRKF and UUOF. (**c**) Quantitative flow cytometric analysis of the cell-surface expression of FGFR1, FGFR, FGFR2, FGFR3 and FGFR4 in NRKF and UUOF. (**d**) Representative Western blot analysis of FGFR1-4 and co-receptor α and b Klotho (Kl) protein expression in UUOF and NRKF compared to rat tissue levels. β-tubulin was used as a loading control. (**e**) Comparative acute effects (30 min) of exogenous FGF23 (1 ng/mL) on signal transduction pathways in NRKF and UUOF transiently transfected with specific transcription factor responsive luciferase constructs. Results are expressed relative to vehicle-treated cells (dotted line). Pathways examined were early growth response 1 (Egr1), MAPK/ERK pathway (serum response element; SRE), MAPK/JNK (AP-1), PI3K/Akt (FOXO), Ca^2+^/PKC (NFAT), TGF-β (Smad), Wnt (T-cell factor; TCF). (**f**) Relative changes in Smad activation (TGF-β bioactivity) in HEK293 cells treated with media from NRKF and UUOF conditioned by exposure to FGF23 (1ng/mL for 24 h) or vehicle (dotted line). Data are shown as mean ± SD and represent at least three independent experiments with similar results. *P<0.05; **P < 0.01; ***P < 0.001; ns, not significant. P-values were determined using Student’s unpaired two-tailed t-test.

Although qualitatively similar, the effect of TGF-β1 on NRKF appeared less than in UUOF cells with respect to enhancement of αSMA/pSmad2/3 staining and the magnitude of mRNA changes in profiling. To corroborate, we used a dual-luciferase reporter assay to test the response to TGF-β1 in each cell type (Fig. 6a). The response in Smad pathway induction in UUOF over vehicle-treated cells was 3-fold higher than that seen in NRKF (60-fold and 20-fold respectively, p < 0.05) (Fig. 6a), consistent with UUOF being hyper-responsive to exogenous TGF-β1. To explain, we looked to see whether altered expression of the TGFBR might account for the increased TGF-β1 bioactivity. As anticipated, flow cytometric analysis of receptor abundance showed that cell-surface expression of TGFBR2 was almost 7-fold higher in UUOF than NRKF, while TGFBR1 was increased 3-fold (Fig. 6b). Interestingly, UUOF had higher levels of cell surface FGFR1 and FGFR4 but lower levels of FGFR3 compared to NRKF (Fig. 6c). These results were confirmed by western blotting (Fig. 6d). Although FGFR2 was present at similar low levels in NRKF and UUOF, αKL was expressed in NRKF but absent in UUOF. βKL, a co-receptor for endocrine FGF19 and FGF21 in the liver^38^, was not present in either cell type (Fig. 6d).

Given the divergent effects of FGF23 on TGF-β1 signalling in NRKF and UUOF, we next sought to determine if there was a downstream difference in the signalling pathways activated (Fig. 6e). Using specific dual-luciferase reporter assays, we found that while FGF23 activated canonical FGFR signalling pathways (MAPK and PI3K/Akt) in NRKF, as in αKL-expressing HEK293 cells (see Supplementary Fig. S5), treatment with FGF23 activated NFAT and AP-1/JNK pathways in UUOF (Fig. 6e).

Although FGF23 caused no acute increase in Smad induction (30 min) in either cell type, we have consistently seen an increase in Smad2/3 phosphorylation in UUOF in response to longer treatment (24 h) with FGF23 (Fig. 5). Taken together with a profile of changes in TGF-β1 signalling target expression that is highly indicative of enhanced TGF-β1 activity, we sought to determine whether changes induced by FGF23 were partly due to the enhancement of autocrine TGF-β1 signalling, driving a ‘feed-forward’ induction of its own expression. Since, TGF-β1 is secreted in bound/inactive form (indeed latent TGF-β1 accounts for >98% of total TGF-β1 in FBS), direct measurement of this growth factor in culture media is an unreliable indicator of TGF-β action. Instead, we employed a luciferase reporter assay of canonical Smad signalling as a more sensitive readout of bioactivity. In agreement, we found that conditioned media from FGF23-treated UUOF activated the Smad pathway in HEK293 reporter cells, whereas media taken from NRKF did not (Fig. 6f).

### C-terminal FGF23 fragments have no effect on fibroblast activation

**Figure 7:**
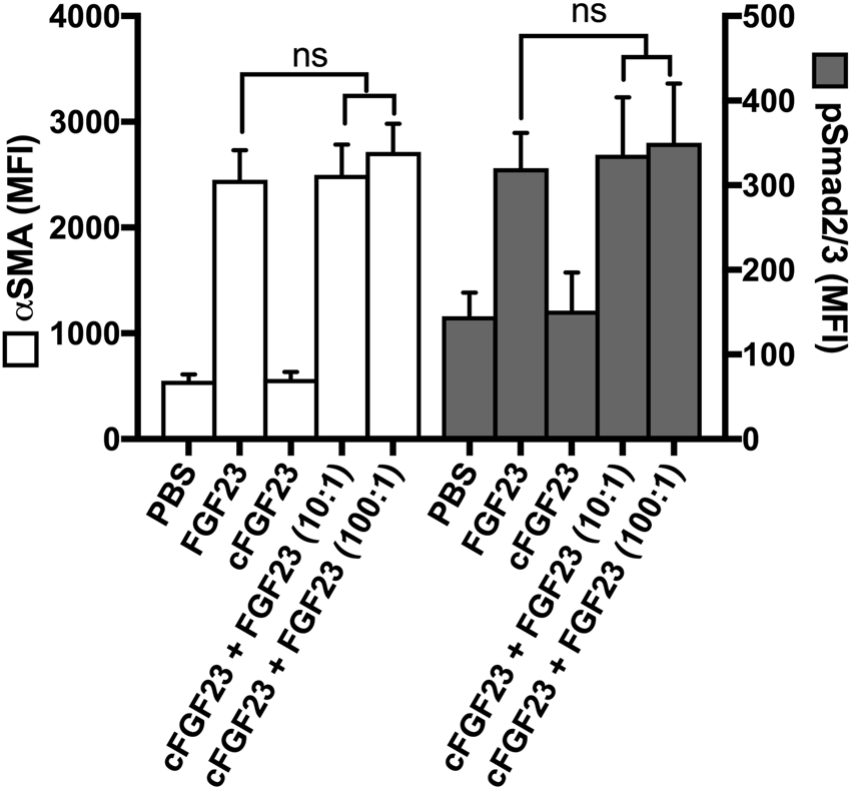
C-terminal FGF23 fragments have no effect on fibroblast activation. Quantitative flow cytometric analysis of intact FGF23 and c-terminal FGF23 (cFGF23), alone or in combination, on changes in αSMA and pSmad2/3levels (mean fluorescence intensity, MFI) in UUOF after 24 h treatment. Data are shown as mean ± SD and are representative of four independent experiments all with similar results. ns, not significant. P-values were determined by one-way ANOVA with Holm-Sidak multiple comparisons test.

The biological activity of cleaved FGF23 fragments remains controversial due to inconsistent reports from experimental studies and lack of supporting evidence from human observational trials, where an excess of C-terminal fragments is not associated with abnormalities in mineral metabolism^39^. Importantly, the pro-fibrotic effects reported here were specific to the intact protein as C-terminal FGF23 fragments had no effect on αSMA and pSmad2/3 levels in UUOF (Fig. 7). Indeed, cFGF23 had no effect on the induction of 35 different signalling pathways in a αKL-expressing HEK293 reporter cell line (see Supplementary Fig. S5), although we acknowledge effects may have been seen at higher molar concentrations not tested here. Moreover, co-administration with intact FGF23, even with C-terminal FGF23 present in 100-fold molar excess, had no effect (Fig. 7), demonstrating that the isolated C-terminal FGF23 fragment is unable to appreciably antagonise the fibrogenic potential of the intact protein in this *in vitro* system.

### Effects of FGF23 on UUOF are FGFR-dependent and partly mediated via TGF-β1 signalling

**Figure 8:**
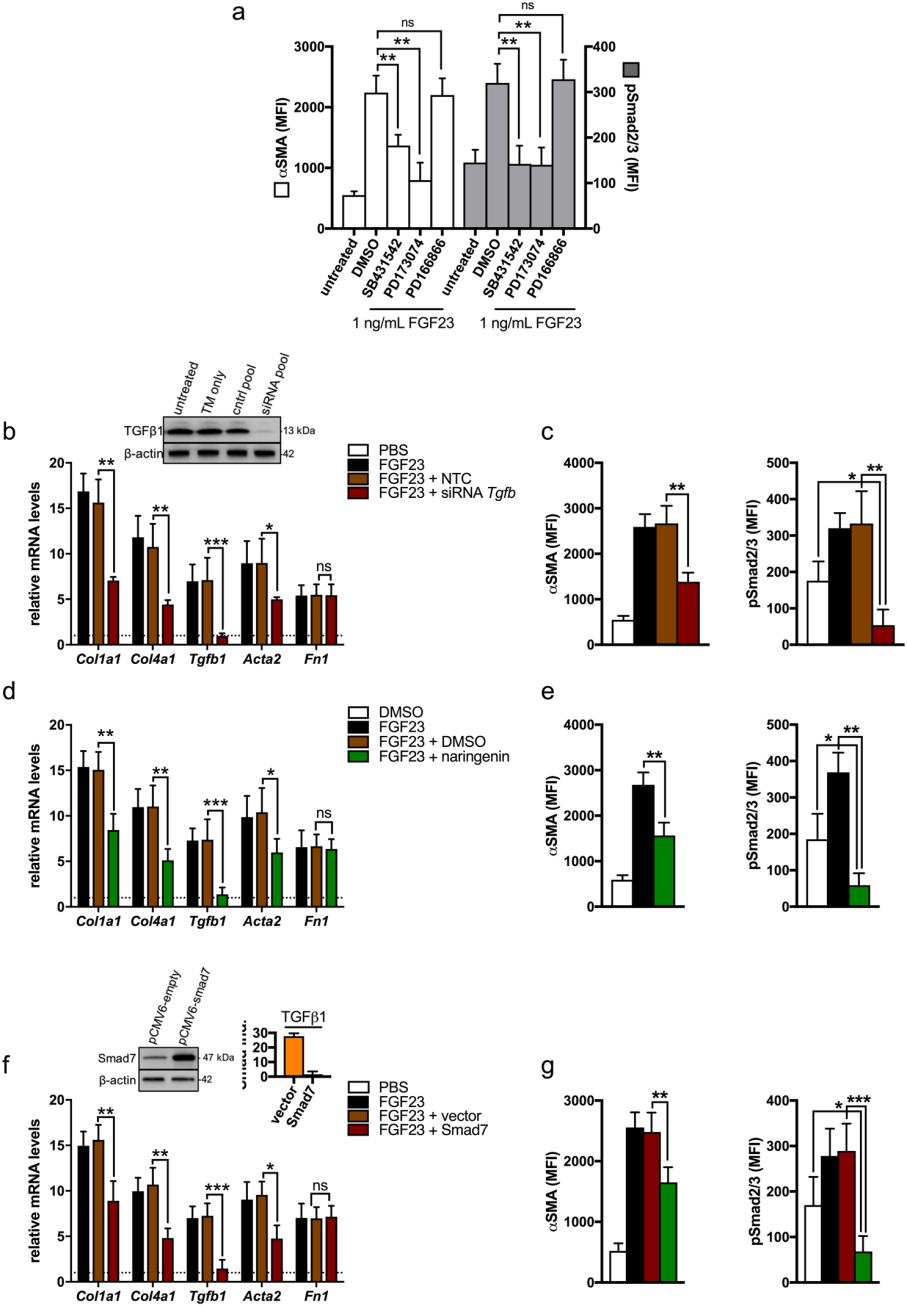
Effects of FGF23 on UUOF are mediated by FGFR and activation of TGF-β1 signalling. (*a*) The effect of pre-treatment with SB431542 (TGFBR1 inhibitor), PD173074 (pan FGFR inhibitor), PD166866 (selective FGFR1 inhibitor), or DMSO (vehicle) on FGF23-induced changes (1 ng/mL for 24 h) in αSMA and pSmad2/3, as analysed by quantitative flow cytometry. (**b**,**c**) The effect of *Tgfb1* specific siRNAs on fibrogenic gene expression using qRT-PCR (**b**), αSMA levels and pSmad2/3 protein levels (**c**) in UUOF treated with FGF23 (1 ng/mL for 24 h), compared to a non-targeting control (NTC) pool. Representative Western blot of TGF-β1 silencing shown above. (**d**,**e**) The effect of pre-treatment with naringenin (Smad 3 inhibitor) on fibrogenic gene expression using qRT-PCR (**d**), αSMA levels and pSmad2/3 protein levels (**e**) in UUOF treated with FGF23 (1 ng/mL for 24 h), compared to a NTC pool. (**f**,**g**) The effect of transient overexpression of Smad7 (endogenous Smad inhibitor) on fibrogenic gene expression using qRT-PCR (**f**), and quantitative flow cytometric analysis of αSMA levels and pSmad2/3 (**g**) in UUOF treated with FGF23 (1 ng/mL for 24 h), compared to cells transfected with empty vector. Representative Western blot (upper left ‘**f**’) of Smad7-transfected UUOF. Suppression of Smad signalling was confirmed using a reporter assay as previously described (upper right ‘**f**’). Data are shown as mean ± SD and represent at least three independent experiments with similar results. *P < 0.05; **P < 0.01; ***P < 0.001; ns, not significant. P-values were determined by one-way ANOVA with Holm-Sidak multiple comparisons test.

Consistent with the effects of FGF23 being FGFR-dependent, pre-treatment of UUOF with PD173074 (a pan-FGFR inhibitor) ameliorated the effect on FGF23-mediated induction of αSMA and pSmad2/3 levels (Fig. 8a). Conversely, a selective FGFR1 inhibitor (PD16686) had no effect on either readout (Fig. 8a).

The observation that exogenous intact FGF23 increased TGF-β1 expression/Smad induction in UUOF led us to postulate that FGF23’s pro-fibrotic effects may be secondary to an autocrine effect via up-regulation of TGF-β1. To test our hypothesis, we evaluated the effect of intact FGF23 on fibrogenesis in cells in which TGF-β1 expression was silenced using siRNA. Here, siRNAs against TGF-β1 partially suppressed FGF-23-induced increases in the expression *Tgfb1*, *Col1a1* and *Col4a1*, but not *Fn1* (Fig. 8b), and reduced protein levels of *a*SMA to below control levels (Fig. 8c). Neither transfection reagent in isolation, nor a non-targeting control, had any effect on FGF23’s pro-fibrotic actions. Furthermore, consistent with effects being partly mediated by the TGF-β1 receptor complex, selective inhibition of the TGFBR1 with SD431542 yielded similar results (Fig. 8a). Blockade of canonical (Smad-dependent) TGF-β1 signalling with naringenin (a Smad 3 inhibitor) (Fig. 8d,e), or transient over-expression of Smad7 (Fig. 8f,g), an endogenous TGF-β signalling antagonist, also had a suppressive effect on FGF23 induced fibrogenesis, but did not block myofibroblast activation altogether. Taken together, these data indicate that FGF23 enhances fibrogenesis in UUOF partly via TGF-β1signalling, downstream of FGFR activation.

## Discussion

Although bone is the principal source of circulating FGF23^24^, controversy surrounds low-level expression in various organs, including the kidney, and its significance. Several studies now suggest that FGF23 has effects beyond its classical mineral handling actions^21, 22^. In the present study, we have identified temporal and spatial changes in renal FGF23 expression in response to acute UUO. Furthermore, we have demonstrated actions of FGF23 *in vitro*, consistent with a pro-fibrotic role, mediated in part by the enhancement of TGF-β1 signalling. Critically, just as the unobstructed C/L kidney does not fibrose in UUO, FGF23 failed to activate fibrogenic pathways in fibroblasts derived from normal kidneys (NRKF). This highlights the need for an initial injury-related signal, to prime the fibroblast, enhance its responsiveness to fibrogenic cues, and potentiate the profibrotic effects of FGF23 in this microenvironment.

That renal disease is not a prominent feature of patients with hereditary or acquired primary hypophosphataemic disorders, nor seen in transgenic animals over-expressing FGF23^40^, seems consistent with these observations and the requirement for a priming step. While this study elaborates strong mechanistic parallels between the effects of FGF23 excess on the kidney and heart, there is a clear distinction in the requirement for a priming signal, as FGF23 appears sufficient to drive cardiac hypertrophy in wild-type healthy animals without pre-existing injury^21^.

Specific renal actions of FGF23, independent of other mineral metabolites and regulators, are difficult to isolate in CKD due to the effects of uraemia and mineral bone disease. In this context, UUO is the ideal vehicle to examine acute fibrogenic effects of FGF23, given the absence of systemic derangements in bone mineral metabolism and preservation of renal function via the intact C/L kidney. In our study, FGF23 mRNA was expressed in tubules of the obstructed kidney, substantiating that it is both made locally, and upregulated in response to injury. Cortical immunohistohemical staining for FGF23 was found in LTL-negative tubules, and to a lesser extent interstitial myofibroblasts, which was only apparent at higher magnification. Although the absence of co-staining with LTL does not definitively exclude proximal tubules as a source of FGF23 due to the loss of LTL after injury, our LCMD studies show that FGF23 mRNA was expressed in tissue containing transcripts characteristically enriched in distal tubules (Slc12a3, Slc22a13, Wnk1)^34^. Taken together, these data suggest that the major source of FGF23 following injury is the distal tubule. Thus far, in the context of kidney disease, renal expression of FGF23 has only been definitively identified in the obese Zucker rat^29^ and rodent models of polycystic kidney disease^28^. Our findings therefore add to the growing list of situations where ectopic renal FGF23 production occurs, and suggests a broader involvement in renal pathologies in response to varied insults.

In agreement with the current analysis, de Jong *et al*. reported that serum C-terminal FGF23 concentrations doubled in their murine UUO model^41^. In their study, administration of exogenous FGF23 increased expression of the transcription factor EGR1, a readout of classical canonical FGF23 signalling, in the C/L kidney but not the obstructed organ, consistent with a lack of physiological responsiveness in the obstructed kidney following injury. This is likely to relate to the loss of the obligate canonical co-receptor α-Klotho, which is well described in the UUO model^42^, as replicated here. Indeed, despite increased intact FGF23 levels in the obstructed kidney, we found no change in the mRNA expression of classical FGF23 targets, Cyp27b1/24a1 or Npt2a/b, although we cannot exclude regulatory changes at the post-transcriptional level^43^. On the other hand, seemingly at odds with our findings, de Jong *et al*. found that FGF23 administration did not exacerbate fibrosis. However, we note a number of important differences. Our own data suggests that the pre- administration prior to UUO, as performed here, may actually suppress myofibroblast activation. The increase in circulating FGF23 was relatively modest, with an unknown bioavailability due to the inevitable poor perfusion of the obstructed kidney and impaired tubular uptake^44^. Most importantly, our study describes a local action of FGF23 contingent on the juxtapositioning of local synthesis and signalling. This may be quite different from actions of circulating FGF23.

In addition to regulating mineral handling, the kidney has an essential role in FGF23 metabolism and is the only organ to extract the hormone from the circulation. Since functionality is compromised in the obstructed kidney of the UUO model, the C/L undergoes various haemodynamic, structural and functional adaptations to persevere clearance and maintain homeostasis^40, 45, 46^. Accordingly, the circulating concentrations of phosphate, calcium, as well as calcitriol, PTH and intact FGF23 were unchanged by UUO. An isolated increase in serum C-terminal FGF23 concentration was observed, consistent with previous studies^41^. On the tissue level, intact bioactive FGF23 was only detected in the obstructed kidney, whereas the isolated C-terminal FGF23 fragment was increased in the C/L kidney, in keeping with changes in renally-expressed proprotein convertases, furin and PC7, which were highest in this organ. In the context of UUO, we speculate that the C/L kidney may cleave the excess intact FGF23 generated by the obstructed kidney via upregulation of the proteolysis machinery, to achieve overall mineral balance, and accounting for the elevated C-terminal FGF23 concentrations but otherwise normal mineral metabolite levels. Given the purely observational and associative nature of these findings, further mechanistic studies are needed to confirm this hypothesis.

Tubulointerstitial fibrosis is driven through multiple intersecting pathways by the recruitment and activation of interstitial fibroblasts by TGF-β1 and other cytokines^32^. The juxtapositioning of tubular FGF23 and interstitial myofibroblasts in the obstructed kidney, as well as that potentially synthesised by myofibroblasts themselves, provided a rationale for examining the effect of FGF23 on fibrogenesis more specifically. To reproduce the *in vivo* situation, we examined fibroblasts propagated from the kidneys of normal rats (D0) and animals 3-days post UUO (D3). Our studies convincingly showed that FGF23 was pro-fibrotic in UUOF, inducing a pattern of changes in gene expression remarkably similar to that seen with TGF-β1. This effect was specific to the full-length FGF23 molecule. However, intact FGF23 did not have the same effect on NRKF, where it may have even potentially supressed fibroblast activation. Furthermore, while both cell types were responsive to TGF-β1, the magnitude of the effect was far greater in UUOF, in line with much earlier studies where it was shown that fibroblasts isolated from fibrotic kidneys were upregulated compared to their normal counterparts^47^.

Accounting for these complex effects is difficult, and unlikely to reflect changes in the regulation of any single factor. When comparing normal and fibrotic fibroblasts, we found that cell-surface expression of receptors for both FGF and TGF-β pathways differed both quantitatively and qualitatively, suggesting that dysregulation of signalling at the level of the receptor may at least partly underpin differences in the responsiveness to these growth factors and their effective bioactivity. Of note, while pre-treatment with a pan-inhibitor of FGFR signalling blocked FGF23 induction of myofibroblast differentiation and TGF-β-signalling in UUOF, pre-treatment with a selective FGFR1 inhibitor had no effect on FGF23-indcued fibrogenesis suggesting that signalling is through other FGFR isotypes mediated non-canonical activation. Since antagonism of canonical TGF-β signalling only partially ameliorated FGF23s’ effects, other TGF-β-independent pathways of fibroblast activation are likely to be involved. Further work is needed to examine the significance of these receptor differences in downstream signalling events and identify the pathways responsible for the transduction of FGF23 pro-fibrotic actions in UUOF.

Cross-talk between FGF and TGF-β-related pathways appears important in the regulation of cellular differentiation, both physiologically (e.g. in osteogenesis), and pathologically (e.g. in atherosclerosis). Interestingly, canonical FGFs (e.g. FGF2) are reported to have similarly divergent effects on TGF-β signalling in endothelial cells and the endothelial-to-mesenchymal transition (endoMT), depending on their tissue of origin and phenotype^48^. These data therefore seem to parallel our own findings with context- and temporally-specific effects of FGF on TGF-β1 signalling.

For the transduction of mineral-regulating signals in the kidney and parathyroids, FGFRs partner with membrane-bound αKL to achieve high-affinity and tissue-specific binding to circulating FGF23. In contrast, pathological FGF23 signalling in the heart occurs independent of αKL, as this receptor is not expressed by the cardiomyocyte^21^. Although UUOF also lack αKL, and hence pro-fibrotic signalling likewise occurs in an αKL-independent manner, membrane-bound αKL was detectable in NRKF. It is unclear whether the absence of αKL in the former is due to a loss if αKL, or derivation from αKL negative cells. Others have shown that the presence of αKL appears to be an important determinant of downstream signalling^22^.

Controversy surrounds the significance of the C-terminal fragment, with original studies indicating that proteolysis abolished phosphaturic effect^49^ and other studies showing that some C-terminal fragments retain the phosphaturic activity of intact hormone^50^. More recent work has suggested that it is in fact a competitive, but non-activating, ligand (i.e. inhibitor) for FGF23 binding to the FGFR-αKL complex^51^. In the present study, the isolated C-terminal fragment of FGF23 showed no direct fibrotic activity in UUOF, nor did it antagonise the pro-fibrotic actions of the intact molecule, even when present in excess. In this context, it is seemingly unrelated to fibrogenesis. Indeed, it should be stressed that inactivation of FGF23 by proteolysis is more consistent with the biochemical picture of patients with excess C-terminal FGF23 but normal intact levels. For instance, phosphate wasting is not a feature of iron deficiency where there are often marked elevations (10-fold) in C-terminal FGF23 but intact levels of the hormone and phosphate remain within the normal range^39^. The absence of (myo)fibroblasts, the fact that FGF23 is cleaved *in situ*, possibly by furin and PC7, to produce inactive C-terminal fragments, and the lack of fibrogenic effects of FGF23 on NRKF, together explain why the C/L kidney does not fibrose.

Our experimental findings are not without limitations. Our findings will need to be confirmed in situations where vascular and glomerular pathologies predominate. Likewise, the lack of uraemia in this model, while in some ways useful, doesn’t recapitulate the systemic features of CKD. Nonetheless, while we are cautious to extrapolate our findings to CKD, we note that several observations made here may inform our understanding of this condition. The experimental downregulation of cleavage enzymes concomitant with the accumulation of intact FGF23 in the injured kidney following UUO, may help to explain the increase in the ratio of intact-to-C-terminal FGF23 observed clinically with progression of CKD^3^, where there is no functioning C/L kidney to moderate FGF23 bioactivity. That FGF23 is only activating towards UUOF, injury-primed fibroblasts, also seems broadly consistent with the epidemiological evidence, which shows that risk estimates for CKD differ depending on the stage of disease. In a cohort of 13,000 healthy adults, Rebholz *et al*. have reported that higher serum FGF23 were strongly associated with progression to ESRD, but had marginal predictive value for incident CKD^10^, suggesting that FGF23 might be more closely related to progression than initiation of disease. Our studies therefore seem to provide a useful correlate of the *in vivo* situation. While our findings in acute injury will also need to be examined in more chronic settings, it is noteworthy that rapid changes in FGF23 have been reported in acute kidney injury (AKI), preceding changes in filtration^12^. Indeed, peri-AKI levels of circulating FGF23 appear predictive of progression to CKD and other complications^13^. Finally, while our observational animal data is consistent with a role for FGF23 in enhancing nascent tubulointerstitial fibrosis, this will need to be experimentally corroborated in studies where specific antagonism of FGF23 signalling is employed.

In conclusion, despite numerous studies independently correlating high FGF23 levels with decline in renal function and progression to ESRD, evidence of a direct mechanism linking the two has remained elusive. These studies confirm renal FGF23 synthesis in the injured kidney, which may augment myofibroblast activation and fibrogenesis locally via activation of TGF-β-related pathways. FGF23 therefore joins a growing list of cytokines and growth factors that potentially regulate renal fibrosis. These actions are specific to the full-length intact molecule, and not antagonised by C-terminal FGF23 fragments. Taken together these findings suggest a role for FGF23 in kidney disease outside of the mineral bone disease entity and strengthen the rationale for therapeutic targeting of off-target FGF23 effects in CKD.

## Methods

### Animal model

Male C57Bl6 were obtained from Monash Animal Research Platform (Melbourne, Victoria, Australia). UUO was performed in mice (age 8–10 weeks) under inhalational general anaesthesia (Methoxyflurane, Abbott, Sydney, NSW, Australia) as previously described^52^. At day 3 (D3) post-UUO, animals were sacrificed by anaesthetic overdose and tissue (obstructed/contralateral kidney and hind limb bone) harvested and portioned for immunohistochemical, protein and RNA analyses. Blood was also collected for biochemical analysis. A parallel control group consisted of tissue and blood taken from un-operated animals (D0). These experiments were approved by the Monash University Institutional Animal Ethics Committee, which adheres to the *Australian Code of Practice for the Care and Use of Laboratory Animals for Scientific Purposes*.

### Growth factors, inhibitors and antibodies

Recombinant human transforming growth factor β1 (TGF-β1) was purchased from PeproTech (#100-21; Rehovot, Israel). Recombinant mouse FGF23 (#2629-FG), and recombinant FGF2 (#3139-FB) were obtained from R&D Systems (Minneapolis, MN, USA). Purified recombinant C-terminal FGF23 fragment, Ser180-Val251, was generated as a custom synthesis by Genscript (Piscataway, NJ, USA). Inhibitors used, and their final concentrations are listed in Supplemental Table S1. Primary and secondary antibodies used are listed in Supplemental Table S2 and Supplemental Table S3 respectively. Validation data for the FGF23 antibodies used in this study is provided in Supplemental Fiugre S6.

### Immunofluorescence staining

For co-staining of FGF23 and Lotus tetragonolobus lectin (LTL), parformaldehyde fixed sections were dewaxed, boiled under pressure in citrate buffer (pH 6.0), blocked with 10% goat serum (Vector Laboratories) in 3% BSA/PBS (pH 7.6) containing 0.1 M glycine for 1 h at RT. Slides were then incubated with rat monoclonal anti-mouse. FGF23 and fluorescein labelled-LTL lectin (FL-1321; Vector Laboratories). For co-staining of FGF23 and αSMA, parformaldehyde fixed sections were subjected to antigen retrieval and blocking as above before sequential incubation with mouse monoclonal anti-αSMA and then with rat monoclonal anti-mouse Since FGF23 is a low-abundance target, signal was amplified using Alexa Fluor 488 or 595 tyramide reagent (Alexa Fluor™ Tyramide SuperBoost™ Kit, streptavidin; Life Technologies) according to the manufacturer’s instructions. Sections were mounted in hard set Vectashield (Vector laboratories). Low power images were taken using a Zeiss AXIOSKOP2 microscope. High power images were visualised on a Leica SP5 confocal microscope with a 63x oil objective. Post-acquisition processing was performed using Fiji- ImageJ (NIH).

### Laser capture microdissection

Laser Capture Microdissection (LCMD) was used to isolate specific nephron segments from fixed paraffin embedded tissue sections using the Veritas™ instrument and Arcturus reagents (Mountain View, CA, USA) as previously described^53^.

### Biochemical studies

Biochemical analysis was performed on serum using standard commercially available assays as described in the Supplemental methods. All samples were run in duplicate in assays with in-house within-run analytical CVs < 7%.

### Proprotein convertase activity assay

Proprotein convertase activity was measured in kidney homogenates using previously published protocols^54^ with minor modifications as detailed in the Supplemental methods. Activity was expressed relative to D0 samples. Human recombinant furin (#F2677; Sigma) was used as a positive control in each run.

### Fibroblast culture and treatments

Primary cultures of fibroblasts propagated from normal kidneys (NRKF) and fibrotic kidneys (3 days after UUO; UUOF) of Sprague-Dawley rats were utilised for these studies, as described before^55^. Cultures were maintained in Dulbecco’s modified Eagle Medium (DMEM) supplemented with 10% foetal bovine serum (FBS), 2.2% HEPES, 1% L-glutamine, penicillin (50 U/mL) and streptomycin (50 μg/mL) in a humidified incubator at 37 °C and 5% CO_2_. For experimental work, fibroblasts were typically cultured for a further 24–48 h in maintenance growth medium before switching to FBS-reduced media (1% FBS) for 24 h before all *in vitro* experiments.

Our experimental approach was to look for effects of FGF23 (diluted in PBS) on fibroblast activation through changes in signal transduction (30 min), mRNA expression of fibrogenic mediators (24 h) and at the protein level (48 h) compared to vehicle treatment (PBS). mRNA and protein targets were selected to match those studied *in vivo*. As a comparator, we treated cells with 1 ng/mL recombinant human TGF-β1, a dose previously shown to maximally induce myofibroblast differentiation. In some experiments cells were pretreated with receptor inhibitors or cell-permeable inhibitors of signalling mediators (or vehicle) at the concentrations stated in the text for 30–60 min (as indicated) before the addition of FGF23.

### Immunocytochemistry

Co-staining with αSMA and vimentin was performed as previously described^56^. Images were captured with a Leica SP5 confocal microscope using a 63x oil immersion objective.

### Cell transfection and luciferase reporter assays

Transient gene silencing in UUOF was performed using pre-designed siRNA (13 nM) ON-TARGET plus SMARTpool reagents from Dharmacon (GE Life Sciences): Tgfb1 (L-091428-02) and a non-targeting control pool (D-001810-10). Fibroblasts were transfected with siRNA using Viromer blue (Lipocalyx) according to the manufacturer’s protocol in FBS-reduced media (1%) with antibiotics for 48 h. In some experiments, UUOF were transiently transfected with plasmids encoding a haemagglutinin (HA)-tagged Smad7. Fibroblasts were transfected with 5 μg of plasmid DNA (per 10^6^ cells) using the Viromer yellow reagent (Lipocalyx) according to the manufacturer's protocol. The Smad7 construct was a gift from Jeff Wrana (Addgene plasmid #11733^57^).

For reporter assays, fibroblasts were transiently transfected with inducible firefly luciferase constructs under the control of specific transcriptional response elements: Egr1 (#CCS-8021L), SRE (#CCS-010L), AP-1 (#CCS-011L), FOXO (#CCS-1022L), NFAT (#CCS-015L), Smad (#CCS-017L) and TCF (#CCS-018L) (all from Qiagen). In each case, co-transfections were performed with 100 ng of reporter plasmid, mixed with 10 ng of a CMV promoter-driven *Renilla* luciferase plasmid (Promega) using Viromer yellow as described above. In some experiments, firefly reporter and Renilla luciferase control were mixed with expression plasmids (350 ng). 48 h post transfection, cells were switched to 0.5% FBS-DMEM for 6 h and then treated with agonists or vehicle (PBS) in fresh serum-reduced media, typically for 30 min, before luminescence measurements were made. Quantification of luciferase activity was performed using the Dual-Glo Luciferase Assay System (#E2920, Promega), according to the manufacturer’s protocol. Renilla luciferase activity was used to control for transfection efficiency and normalise firefly luciferase activity. Normalised luciferase activities were determined in triplicate and expressed as fold increase relative to basal activities measured in control vector-transfected cells or vehicle-treated controls.

HEK293 (ATCC; #CRL-1573) were cultured in DMEM supplemented with 10% FBS, GlutaMAX (2 mM; #35050061, Thermo Scientific), penicillin (50 U/mL) and streptomycin (50 μg/mL) in a humidified incubator at 37 °C and 5% CO_2_. Transient transfections in HEK293 cells were performed using the Lipofectamine 3000 reagent (Life Technologies) according to the manufacturer’s protocol. The plasmid construct encoding V5-tagged membrane Klotho was a gift from Hal Dietz (Addgene plasmid #17712^58^). Signal transduction pathways were screened using a Cignal 45-Pathway Reporter Array from Qiagen (#CCA-901L) and were performed according to the manufacturers instructions.

### RNA isolation and qRT-PCR

RNA was extracted using the Qiagen miRNeasy Mini kit according to manufacturer’s instructions. 1 μg of RNA was reverse-transcribed using the iScript RT supermix kit (Bio-Rad). Quantitative real-time PCR (qRT-PCR) was performed in triplicate in a CFX96 cycler (Bio-Rad) using equal volumes of cDNA and the SsoAdvanced™ Universal SYBR Green Supermix (Bio-Rad) with custom-made gene-specific primers (Sigma-Aldrich) or using validated PCR Prime assays (Bio-Rad) (Supplemental Table S4). The mRNA level of target genes was normalised to the reference gene, β-actin, and expressed relative to an appropriate control (D0 or vehicle-treated) using the 2^−ΔΔCt^) method.

For LCMD tissue, RNA extraction and purification was performed using the Paradise PLUS Reagent System (Arcturus) according to the manufacturer’s instructions with modifications as described previously^53^.

### Western blot analysis

Western blotting studies were performed as previously described^59^. Proteins were resolved on 10% Mini-PROTEAN TGX stain-free pre-cast gels (Bio-Rad) and transferred onto PVDF using the Trans-Blot Turbo transfer system. Membranes were probed with specific primary antibodies against proteins of interest overnight at 4 °C diluted in appropriate blocking buffer followed by incubation with a HRP-conjugated secondary. Membranes were developed in SuperSignal West Pico PLUS Chemiluuminescent substrate (#34580, Thermo Scientific) and imaged using ChemiDoc Imaging System (BioRad). Data are presented as fold change in protein expression relative to control groups (D0 animals or vehicle-treated cells) after normalisation to loading controls (β-tubulin). Total protein was used to assess equal loading in studies of different tissues using the stain-free system (BioRad).

### Flow cytometric analysis

Cell surface and intracellular staining were performed according to standard protocols for indirect detection as described in the Supplemental methods. For all analyses, a minimum of 10,000 events were acquired at high flow rate, gating on live cells. Flow cytometry was performed using a BD FACSVerse system and data acquired using BD FACSuite software. Raw data was imported into FlowJo LLC (Ashland, Oregon) for analysis. Data are presented as mean fluorescence intensity (MFI) or fold change when compared to vehicle (PBS/DMSO) treatment.

### Statistics

Results were analysed with GraphPad Prism 7.0 (La Jolla, CA, USA). All data are presented as the mean ± SD with two-tailed P < 0.05 defined as statistically significant.

## Electronic supplementary material

Supplementary material
