# Supplementary material for: FGF23 is synthesised locally by renal tubules and activates injury-primed fibroblasts
Source: Sci Rep. 2017 Jun 13;7:3345. doi: 10.1038/s41598-017-02709-w (PMC5469734; doi:10.1038/s41598-017-02709-w)
Supplement: Supplementary file 1 — Supplementary material [file 41598_2017_2709_MOESM1_ESM.pdf]

# SUPPLEMENTARY INFORMATION

**for**

# FGF23 is synthesised locally by renal tubules and activates injury-primed fibroblasts

Edward R. Smith[1,2*], Sven-Jean Tan[1,2], Stephen G. Holt[1,2], Tim D. Hewitson[1,2]

[1]Department of Nephrology, The Royal Melbourne Hospital, Melbourne, Victoria, Australia

[2]Department of Medicine- Royal Melbourne Hospital, University of Melbourne, Melbourne, Victoria, Australia.

*Corresponding author
Dr. Edward R. Smith
Department of Nephrology
The Royal Melbourne Hospital
Parkville Vic 3050
Australia
Email: edward.smith@mh.org.au

## SUPPLEMENTARY METHODS

### *Animal model*

Unilateral ureteric obstruction (UUO) is a rapid and reproducible model of primary tubulointerstitial fibrosis, occurs independently of species and strain and demonstrates changes that mimic the pathology of human progressive CKD [1]. Male C57Bl6 were obtained from Monash Animal Research Platform (Melbourne, Victoria, Australia). UUO was performed in mice (age 8-10 weeks) under inhalational general anaesthesia (Methoxyflurane, Abbott, Sydney, NSW, Australia). The left ureter of animals was ligated with 5.0 surgical silk, while the contralateral ureter was left intact. The incision was sutured and mice allowed to recover with Temgesic (Burprenorphine; Reckitt Benckiser, West Ryde, NSW, Australia) administration. Animals were housed in a controlled environment with normal dark-light cycle and free access to water and rodent lab chow (Barastock Stockfeeds, Pakenham, Victoria, Australia).

At day 3 (D3) post-UUO, animals were sacrificed by anaesthetic overdose and tissue (obstructed/contralateral kidney and hind limb bone) harvested and portioned for immunohistochemical, protein and RNA analyses. For protein/RNA studies tissue was snap-frozen in liquid nitrogen and stored at -80°C until extraction. For IHC analysis, portions of kidney were fixed in 4% paraformaldehyde or methyl caronys and processed for paraffin embedding. Blood was also collected for biochemical analysis by cardiac puncture and supplemented with a broad-spectrum protease inhibitor cocktail (EDTA-free; Sigma) to limit ex vivo FGF23 degradation [2]. Serum was obtained by centrifugation (3000 *g* 15 min) and stored at -80°C for batched analysis. A parallel control group consisted of tissue and blood taken from un-operated animals (D0). These experiments were approved by the Monash University Institutional Animal Ethics Committees, which adhere to the Australian Code of Practice for the Care and Use of Laboratory Animals for Scientific Purposes.

***Growth factors, Inhibitors and antibodies***

Chemicals were analytical grade and purchased from Sigma (St. Louis, MO, USA) unless otherwise stated. Recombinant human transforming growth factor β1 (TGFβ1) was purchased from PeproTech (HEK293-derived; #100-21; Rehovot, Israel). Recombinant mouse FGF23 (NS0-derived 6-His-tagged Tyr25-Val251 [Arg179Gln]; #2629-FG), harbouring the R179Q proteolysis-resistant mutation [3], and recombinant FGF2 (E. coli-derived, Ala11-Ser154; #3139-FB) were obtained from R&D Systems (Minneapolis, MN, USA). Purified recombinant C-terminal FGF23 fragment, Ser180-Val251 (N-terminal His-tagged, expressed in E. coli), was generated as a custom synthesis by Genscript (Piscataway, NJ, USA). We confirmed the biological activity of the recombinant mouse FGF23 by demonstrating a specific dose-dependent increase in Erg1 and SRE luciferase reporter activity in HEK293 cells transiently expressing V5-tagged membrane αKl (Supplementary Fig. S5). This is consistent with previous reports by other authors [4, 5], which have shown this source of recombinant protein to have biological activity in vitro (Erg1/Erk induction) and in vivo (i.e. phosphaturic).

Inhibitors used, and their final concentrations are listed in SupplementaryTable S1. None of the compounds were found to significantly reduce cell viability (MTT assay) over 24h, when used at the stated concentrations. Primary and secondary antibodies used are listed in Supplementary Table S2 and Supplementary Table S3 respectively.

***Immunoperoxidase staining***

Adjacent portions of renal tissue were fixed in methyl carnoys or 4% paraformaldehyde in phosphate buffered saline (PBS), processed and embedded in paraffin wax. For DAB immunohistochemistry, tissue sections were dewaxed in xylene, rehydrated in graded alcohol, endogenous peroxidase activity was quenched using 3% $H_2O_2$ in water for 30 min, before non-specific binding sites were blocked with 10% normal horse serum for a further 30 min at RT

(Vector Laboratories, Burlinghame, CA, USA). Sections were then incubated with rabbit polyclonal anti-collagen I (methyl caronys) or rabbit monoclonal anti-vimentin (4% paraformaldehyde). In each case, antibody binding was visualised using an avidin-biotin-complex (ABC Elite; Vector Laboratories) and 3,3'-diaminobenzidine (DAB; Sigma). For detection of α -smooth muscle actin, parformaldehyde fixed sections were prepared as above and then incubated with mouse monoclonal αSMA, conjugated to biotin (ARK kit; Dako), and incubated with streptavidin peroxidase (ARK) and DAB. Finally sections were counterstained with haematoxylin (Dako), dehydrated, and mounted in DePex (Merck) [6]. Images were taken using an Olympus BX50 microscope using 10x magnification.

***Immunofluorescence staining***

For immunofluorescent co-staining of FGF23 and Lotus tetragonolobus lectin (LTL), parformaldehyde fixed sections were boiled under pressure in citrate buffer (pH 6.0), equilibrated in PBS for 30 min and free aldehyde groups reduced by immersion in $NH_4Cl$ (50 mmol/L) in PBS for 20 min at room temperature. Non-specific binding sites were blocked with 10% goat serum (Vector laboratories) in 3% BSA/PBS (pH 7.6) containing 0.1 M glycine for 1h at RT. Slides were then incubated with rat monoclonal anti-mouse FGF23 in 1% BSA in PBS overnight at 4°C. Sections were then incubated with biotin-conjugated goat anti-rat IgG (Vector Laboratories) and fluorescein labelled-LTL lectin (1:200; FL-1321; Vector Laboratories) in 1% BSA in PBS for 2h at RT. DAPI (2 µg/mL in PBS, 15 min, RT) was used a nuclear stain. For immunofluorescent co-staining of FGF23 and αSMA, parformaldehyde fixed sections were subjected to antigen retrieval and blocking as above before incubation with mouse monoclonal anti-αSMA in1% BSA/PBS for 2h at RT. Sections were then incubated with rat monoclonal anti-mouse FGF23 in in1% BSA/PBS overnight at 4°C, before the addition of biotinylated-anti-rat IgG secondary and Alexa Fluor 594 anti-mouse IgG (Life Technologies, Carlsbad, CA, USA) in1% BSA/PBS for 2h at RT. Since FGF23 is a low-

abundance target, signal was amplified using Alexa Fluor 488 or 595 tyramide reagent (Alexa Fluor™ Tyramide SuperBoost™ Kit, streptavidin; Life Technologies) according to the manufacturer's instructions. Sections were washed in PBS and mounted in hard set Vectashield (#H-1400; Vector laboratories). Low power images were taken using a Zeiss AXIOSKOP2 microscope using 0.75x or 20x magnification or visualised on a Leica SP5 confocal microscope with a 63x oil objective for high power imaging. Post-acquisition processing was performed using Fiji- ImageJ (NIH).

The mouse FGF-23 antibody (#MAB2629; R&D systems) used here has been employed in the IHC detection of FGF23 in multiple rodent studies [7, 8], including ectopic detection in the kidney [9]. Specificity was confirmed here by blocking studies using antiserum pre-absorbed with a 10 molar excess of the immunising peptide (#2629-FG; R&D systems). After centrifugation, the supernatant was used at the same working dilution, and in parallel with anti-FGF23 staining, as above (Supplementary Fig.S6). Comparison of immunohistochemical detection without amplification using SuperBoost is also shown alongside.

***Laser capture microdissection***

Laser Capture Microdissection (LCMD) was used to isolate specific nephron segments from fixed paraffin embedded tissue sections using the Veritas™ instrument and Arcturus reagents (Mountain View, CA, USA) [10].

Paraffin wax embedded methyl carnoys fixed tissue blocks prepared above were re-sectioned at 5-10μm. Sections were collected on RNase free plastic slides (Arcturus). The slides were de-waxed using RNase-free techniques, and stained using the HistoGene kit (Arcturus) to provide differential contrast for subsequent micro-dissection. HistoGene stain (Arcturus) provides differential staining of nuclei (purple) and cytoplasm (light pink) while preserving RNA integrity. The contrast provided by the HistoGene stain was used to identify

cortical glomerular and tubular profiles for excision. A Veritas Microdissection Instrument (Arcturus), which utilizes the IR capture laser with a UV cutting laser, was used to laser capture a distinct population of glomeruli and tubules. After dissecting individual glomeruli and tubules (>50 per section) from surrounding tissue, each was captured into an Adhesive Cap (Arcturus) using the IR capture laser. After the cells were collected, the cap was immediately transferred to a microfuge tube containing extraction buffer (Arcturus) and processed as detailed below.

***Biochemical studies***

Serum obtained from animals at D0 and D3 post UUO were analysed for creatinine (MAK080; Sigma), urea (MAK-006), total calcium (MAK022; Sigma), phosphate (#DIPI-500; BioAssay systems, Hayward, CA, USA), 1-84 PTH (#60-2305; Immutopics Inc, San Clemente, CA, USA ), 1,25$(OH)_2$ vitamin D (#AC-62F1; Immunodiagnostic Systems, Tyne & Wear, UK), bone-specific alkaline phosphatase (#CSB-E11914m; Cusabio, Wuhan, China), intact FGF23 (#60-6800; Immutopics, Inc) and C-terminal FGF23 (#60-6300; Immutopics, Inc), using commercially available colorimetric assays or ELISA and run according to manufacturer's instructions. Intact FGF23 and C-terminal FGF23 assays were calibrated using the same set of standards supplied by the manufacturer in the intact kit, allowing direct comparison of measurements in pg/mL. All samples were run in duplicate in assays with in-house within-run analytical CVs <7%.

***Proprotein convertase activity assay***

Proprotein convertase activity was measured in kidney homogenates using previously published methods [11] with minor modifications as detailed. Snap frozen tissue was homogenised by Tissuelyser LT (Qiagen, Hombrechtikon, Switzerland) in lysis/reaction buffer (1 mM $CaCl_2$, 5 mM β-mercaptoethanol, 0.5% Triton X-100 in 100 mM HEPES [pH 7.2]),

cleared by centrifugation at 12,000 *g* for 10 min at 4°C, and the supernatant collected for total protein determination (Qubit; Thermo Scientific, Waltham, MA, USA) and assay of enzyme activity. Samples were diluted with reaction buffer to normalise total protein content and then aliquots pretreated with the PC inhibitor, hexa-D-arginine (HDA) at 1 µM or vehicle (PBS) for 30 min at 4°C, before addition into black FluoroNunc 96 well plates (Thermo Scientific), each in triplicate. The reaction was started by the addition of pre-warmed fluorogenic substrate (furin, PC1, PC7), L-PyroGlu-Arg-Thr-Lys-Arg-7-amino-4-methylcourmarin (#ES013; R&D Systems), to a final concentration of 50 µM in a reaction volume of 100 µL. Fluorescence (ex: 380 nm; em: 460nm) was measured at 2 min intervals for 60 min at 37°C in a thermostatically controlled multimode microplate reader fitted with appropriate filters (BioTek, Winooski, VT, USA). PC activity was determined after background subtraction (HDA-treated samples) and regression analysis of the linear portion of the slope using Prism 7 (GraphPad, La Jolla, CA, USA). Activity was expressed relative to D0 samples. Human recombinant furin (#F2677; Sigma) was used as a positive control in each run.

***Fibroblast culture and treatments***

Primary cultures of fibroblasts propagated from normal kidneys (NRKF) and fibrotic kidneys (3 days after UUO; UUOF) of Sprague-Dawley rats were utilised for these studies, as described before [12]. Cultures were maintained in Dulbecco's modified Eagle Medium (DMEM) supplemented with 10% foetal bovine serum (FBS), 2.2% HEPES, 1% L-glutamine, penicillin (50 U/mL) and streptomycin (50 µg/mL) in a humidified incubator at 37˚C and 5% $CO_2$. Cells were characterized immunocytochemically and were positive for the mesenchymal marker vimentin, negative for the epithelial marker cytokeratin, and only occasionally positive for desmin (a marker of mesangial cells and some myofibroblasts). Based on αSMA staining, approximately 20-30% of NRKF and 100% of UUOF were myofibroblasts when cultured in

this basal media (Supplementary Fig.2). For experimental work, cells were seeded into 6-well plates (Costar, Corning, NY, USA) at $1x10^6$ cells/well for qRT-PCR, Western blotting and flow cytometric analyses or in $25cm^2$ flasks (TPP, Trasadingen, Switzerland) at $5x10^6$ cells/flask for mRNA profiling and immunoprecipitation studies. Transfections were performed in 6-well plates seeded at $1x10^6$ cells/well or 24-well plates seeded at $5x10^4$ cells/well. After attachment overnight and removal of floating cells, fibroblasts were typically cultured for a further 24-48h in maintenance growth medium before switching to FBS-reduced media (1% FBS) for 24h before all in vitro experiments.

In general, our experimental approach was to look for effects of FGF23 (diluted in PBS) on fibroblast activation through changes in signal transduction (30 min), mRNA expression of fibrogenic mediators (24h) and at the protein level (48h) compared to vehicle treatment (PBS). mRNA and protein targets were selected to match those studied in vivo. As a comparator, we treated cells with 1 ng/mL recombinant human TGFβ1, a dose previously shown to maximally induce myofibroblast differentiation. In some experiments cells were pretreated with receptor inhibitors or cell-permeable inhibitors of signalling mediators (or vehicle) at the concentrations stated in the text for 30-60 min (as indicated) before the addition of FGF23.

***Immunocytochemistry***

Fibroblasts were seeded onto glass coverslips (#474030-9000, Carl Zeiss) in 6 well plates at low density ($1x10^5$ /well), and treated with $TGFβ_1$ (1 ng/mL), FGF23 (1 ng/mL) or vehicle (PBS) in 5%FCS/DMEM for 48h. After treatment cells were washed with warmed DMEM and fixed with ice-cold methanol (15 min at 4°C). Coverslips were then air-dried, post-fixed in 4%PFA/0.2%TritonX-100 in PBS for 5 min at RT and blocked in 10% normal goat serum/3% BSA/0.1M glycine in PBS for 45 min. For staining, coverslips were incubated

in mouse anti-αSMA (Dako) and rabbit anti-vimentin (Abcam) antibodies diluted in blocking buffer for 2h at RT in a humidified chamber, washed three times in PBS (5 min each), and then incubated in Alexa Fluor-conjugated goat anti-species secondary antibodies (Life Technologies) diluted in blocker for 60 min in the dark at RT. Nuclear staining was performed with DAPI (1 μg/mL diluted in PBS) for 15 min at RT. Finally, coverslips were washed three times in PBS (5 min each) and mounted in hardset Vectashield (Vector Labortatories). Images were captured with a Leica SP5 confocal microscope using a 63x oil immersion objective.

***Cell transfection and luciferase reporter assays***

Transient gene silencing in UUOF was performed using pre-designed siRNA (13 nM) ON-TARGET plus SMARTpool reagents from Dharmacon (GE Life Sciences): rat Tgfb1 (L-091428-02), and a non-targeting control pool (D-001810-10). Fibroblasts were transfected with siRNA using Viromer blue (Lipocalyx) according to the manufacturers protocol in FBS-reduced media (1%) with antibiotics for 48h. Cells were ~60-70% confluent at the time of transfection. Maximal silencing was achieved 48-72h after transfection (confirmed by Western blotting as shown), at which time cells were treated with FGF23 (1 ng/mL) for a further 24-48h before extraction of RNA/protein or flow cytometric analysis. All treatments were performed in triplicate alongside wells treated with diluted transfection media only (i.e. no siRNA).

In other experiments, UUOF were transiently transfected with plasmid DNA encoding haemagglutinin (HA)-tagged Smad7. Fibroblasts were transfected with 5 μg of plasmid DNA (per $10^6$ cells) using the Viromer yellow reagent (Lipocalyx) according to the manufacturers protocol. The Smad7 construct was a gift from Jeff Wrana (Addgene plasmid #11733; [13]). Protein over-expression was confirmed by Western blotting of lysates probed using an anti-HA antibody 24h after gene transfection. Samd7 over-expressing cells were treated with FGF23 (1 ng/mL) for a further 24-48h before extraction of RNA/protein or flow cytometric analysis.

For reporter assays, fibroblasts were seeded into 96-well opaque flat-bottom culture plates ($1.5x10^4$ cells/well) in complete media for 24h and then maintained in reduced-FBS media for a further 24h prior to transfection. Cells were then transiently transfected with inducible firefly luciferase constructs under the control of specific transcriptional response elements: Egr1 (#CCS-8021L), SRE (#CCS-010L), AP-1 (#CCS-011L), FOXO (#CCS-1022L), NFAT (#CCS-015L), Smad (#CCS-017L) and TCF (#CCS-018L) (all from Qiagen). In each case, co-transfections were performed with 100 ng of reporter plasmid, mixed with 10 ng of a CMV promoter-driven *Renilla* luciferase plasmid (Promega) using Viromer yellow as described above. In some experiments, firefly reporter and Renilla luciferase control were mixed with expression plasmids (350 ng). 48h post transfection, cells were switched to 0.5% FBS-DMEM for 6h and then treated with agonists or vehicle (PBS) in fresh serum-reduced media, typically for 30 min, before luminescence measurements were made. In some experiments, cells were pre-treated for 30 min with receptor/signaling inhibitors before the addition of FGF23. Quantification of luciferase activity was performed using the Dual-Glo Luciferase Assay System (#E2920, Promega), according to the manufacturers protocol. Renilla luciferase activity was used to control for transfection efficiency and normalise firefly luciferase activity. Normalised luciferase activities were determined in triplicate and expressed as fold increase relative to basal activities measured in control vector-transfected cells or vehicle-treated controls.

HEK293 cells were obtained from ATCC (#CRL-1573) and were cultured in DMEM supplemented with 10% foetal calf serum (FCS), GlutaMAX (2mM; #35050061, Thermo Scientific) penicillin (50 U/mL) and streptomycin (50 µg/mL) in a humidified incubator at 37˚C and 5% $CO_2$. Transient transfections in HEK293 cells were performed using the Lipofectamine 3000 reagent (Life Technologies) according to the manufacturer’s protocol. The plasmid construct encoding V5-tagged membrane Klotho was a gift from Hal Dietz (Addgene

plasmid #17712; [14]). Signal transduction pathways were screened using a Cignal 45-Pathway Reporter Array from Qiagen (#CCA-901L) and were performed according to the manufacturers instruction. To test for TGFβ bioactivity, HEK293 cells co-transfected with Smad-reporter (#CCS-017L, Qiagen) and Renilla luciferase (Promega) as above, were treated with 0.45 μm-filtered media conditioned by fibroblasts following exposure to FGF23 (1 ng/mL). Filtered conditioned media from vehicle-treated fibroblasts was used to determine basal activity (=1.0).

***RNA isolation and qRT-PCR***

RNA was extracted from homogenised tissue (TissueLyser LT) or cultured cells in 6 well plates using the Qiagen miRNeasy Mini kit according to manufactures' instructions. 1 μg of RNA was reverse-transcribed using the iScript RT supermix kit (Bio-Rad). Quantitative real-time PCR (qRT-PCR) was performed in triplicate in a CFX96 cycler (Bio-Rad) using equal volumes of cDNA and the SsoAdvanced™ Universal SYBR Green Supermix (Bio-Rad) with custom made gene-specific primers (Sigma-Aldrich) designed in Primer3 (NIH) or using validated PCR Prime assays (Bio-Rad) (Supplementary Table S4). PCR conditions were set according to the manufacturer's instructions. Melt curve analysis was performed to verify the purity and specificity of the amplicons. Direct sequencing was used to confirm the specific amplification of FGF23 transcripts in whole kidney lysates and LCMD tissue. Threshold cycles were calculated using CFX Manager Software (Bio-Rad), and the mRNA level of target genes was normalised to the reference gene, β-actin, and expressed relative to an appropriate control (D0 or vehicle-treated) using the $2^{(-\Delta\Delta Ct)}$ method. For detection analyses, PCR products were resolved on 10% TBE pre-cast gels and visualised with SYBR Safe DNA gel stain (Invitrogen, Carlsbad, CA, USA) on a ChemiDoc MP imaging platform (Bio-Rad).

For LCMD tissue, RNA extraction and purification was performed using the Paradise PLUS Reagent System (Arcturus) according to the manufacturer's instructions with modifications as described previously [10]. The quality of the extracted RNA was assessed using the Paradise Quality Assessment kit with the ratio of 3'- and 5'-directed β-actin transcripts <10. First and second strand cDNA was synthesised using Superscript III RT (Invitrogen) and purified, before Whole Transcriptome Amplification (Arcturus) and purification of the amplified RNA. A second round of cDNA synthesis was performed to generate the template for gene expression.

***$RT^2$ Profiler PCR array***

Total RNA was isolated from $25cm^2$ flasks of vehicle-, FGF23- or $TGF\beta_1$-treated cultures of NRKF and UUOF as previously described and reverse-transcribed using the $RT^2$ First Strand Kit (SABiosciences). 84 genes responsive to TGFß signal transduction were assessed by qRT-PCR using the Rat TGFß Signalling Targets $RT^2$ Profiler PCR array (#PARN-235ZD; Qiagen) according to the manufacturer's instructions using the CFX cycler (BioRad). Data from two independent experiments was analysed using a web-based program (SABiosciences, http://pcrdataanalysis.sabiosciences.com/pcr/arrayanalysis.php). Here, the expression level for each gene of interest (GOI) was calculated as 2-Ct followed by normalisation to the most stable reference genes (determined for each experiment using the HKG algorithm), using the formula 2 − (Ct GOI − Ct HKG). Fold change in normalised gene expression was calculated by comparing values from treated vs. vehicle for each cell type according to the formula: 2-Ct FGF23 or $TGFß_1$ treated /2-Ct vehicle.

***Western blot analysis***

Western blotting studies were performed as previously described [15]. For protein extraction from isolated mouse tissue, samples were minced and then homogenised using a

TissueLyser LT (Qiagen) 1:10 (w/v) in ice-cold RIPA (150 mM NaCl, 1.0% IGEPAL® CA-630, 0.5% sodium deoxycholate, 0.1% SDS, 50 mM Tris, pH 8.0; #R0278, Sigma) supplemented with a protease and phosphatase inhibitor cocktail (#78440, ThermoScientific) and 1mM EDTA and 1 mM EGTA. Homogenates were incubated with gentle mixing for 30 min at 4°C. Insoluble debris was removed by centrifugation (12,000 *g* for 20 min at 4°C) and the supernatant reserved for total protein and Western blotting analysis. For whole cell protein analysis, cells were scrapped from 6 well plates in ice-cold RIPA lysis buffer supplemented with protease and phosphatase inhibitor mixture (#78440, ThermoScientific) with gentle mixing for 30 min at 4°C. Lysates were cleared as described above and the total protein concentration was determined by BCA assay (ThermoScientific). Soluble lysates were heat denatured at 95°C for 10 minutes in 4x Laemmli Sample Buffer (BioRad) containing 10% 2-mercaptoethanol (BioRad). Proteins (20 µg/lane) were resolved on 10% Mini-PROTEAN TGX stain-free pre-cast gels (Bio-Rad Laboratories, Richmond, CA, USA), transferred onto PVDF the Trans-Blot Turbo transfer system (Bio-Rad) and blocked in either 5% (wt/vol) non-fat milk in TBS containing 0.1% Tween-20 (TBST) or 5% BSA in TBST for 2h at RT. Membranes were probed with specific primary antibodies against proteins interest overnight at 4°C diluted in appropriate blocking buffer (non-fat milk: FGFR1-4, α/ß klotho, HA, V5; BSA: others), followed by incubation with a HRP-conjugated secondary (Abcam) for 2h at RT also diluted in blocking buffer. Membranes were developed in SuperSignal West Pico PLUS Chemiluuminescent substrate (#34580, Thermo Scientific) and imaged using ChemiDoc Imaging System (BioRad) equipped with Image Lab software for quantification of band signal intensities (Bio-Rad). Data are presented as fold change in protein expression relative to control groups (D0 animals or vehicle-treated cells) after normalisation to loading controls (β-tubulin). Total protein on the blot was used to assess equal loading in studies of different tissues using the stain-free system (BioRad).

To substantiate the specificity of the anti-mouse FGF23 antibody (#MAB2629, R&D Systems) used for our Western blotting studies we analysed purified mouse intact FGF23 and isolated C-terminal FGF23 fragments (Supplementary Fig. S6). Here, bands for intact and C-terminal FGF23 identified in kidney tissue, co-electrophorese with their corresponding purified recombinant intact (#2629-FG) and C-terminal peptides (Genscript). Neither intact nor C-terminal fragments are present in liver (a negative control), or in the kidney when the primary antibody is omitted (a technical control). Incubation of intact FGF23 peptides with furin resulted in the generation of C-terminal FGF23 fragments, but not with ADHR FGF23 (Genscript), where the proprotein convertase $^{176}$RXXR$^{179}$ cleavage site contains missense mutations ($^{176}$QXXQ$^{179}$).

***Flow cytometric analysis***

Cell viability, cell cycle analysis and proliferation (BrdU incorporation, 10 µM 2h post-treatment) were measured using kits from BD Biosciences and performed according to the manufacturer's instructions (#556547 & #557891). Cell surface staining for FGFRs and TGFBRs was performed according to standard protocols for indirect detection. Briefly, fibroblasts were detached from culture vessels using Accutase (Accutase™ Enzyme Cell Detachment Medium; #561527, BD Bioscience), washed, and resuspended in DPBS containing viability dye (Fixable Viability Stain 450; #562247, BD Biosciences) and incubated at RT for 15 min. Cells were then washed in staining buffer (#00-4222, eBioscience) and aliquots ($2x10^5$ cells) incubated with antibodies specific to each receptor/channel for 60 min at 4°C. After washing, cells were resuspended in staining buffer containing fluorochrome-labelled secondary antibodies and incubated for 30 min at 4°C in the dark. Cells were then washed and resuspended in ice-cold staining buffer for analysis. Isotype controls were from Abcam (rabbit monoclonal IgG #ab172730; mouse monoclonal IgG #ab18457; rabbit

polyclonal IgG #ab171870). For αSMA and pSmad2/3 intracellular staining, fibroblasts were detached with Trypsin/EDTA and incubated with viability dye as described above. Cells were fixed with BD CytofixTM fixation buffer (#554655, BD Biosciences), permeabilised with BD PhosflowTM Perm Buffer III (#558050, BD Biosciences), and stained with Alexa Fluor 647 rabbit monoclonal anti-α-smooth muscle actin (Abcam) and PE rabbit monoclonal anti-pSmad2/3 antibodies (Cell Signalling Technology) for 30 min at RT in the dark. Cells were washed in Perm/Wash buffer (BD Biosciences) and analysed alongside cells stained with appropriate isotype controls: Alexa Fluor 647 rabbit monoclonal IgG, (clone EPR25A #ab199093, Abcam) and PE rabbit monoclonal IgG (clone EPR25A, #ab209478, Abcam).

For all analyses, a minimum of 10,000 events were acquired at high flow rate, gating on live cells. Flow cytometry was performed using a BD FACSVerse system and data acquired using BD FACSuite software. Raw data was imported into FlowJo LLC (Ashland, Oregon) for analysis. Data are presented as mean fluorescence intensity (MFI) or fold change when compared to vehicle (PBS/DMSO) treatment.

***Statistics***

Results were analysed by one-way ANOVA with Holm-Sidak multiple comparisons test when comparing 3 or more groups or using a two-tailed unpaired Student's t-test when comparing 2 groups (GraphPad Prism 7.0, La Jolla, CA, USA). All data in this paper are presented as the mean ± SD with $P < 0.05$ defined as statistically significant.

## References

1. Chevalier RL, Forbes MS and Thornhill BA. Ureteral obstruction as a model of renal interstitial fibrosis and obstructive nephropathy. *Kidney Int*. 2009;75:1145-52.
2. Smith ER, Ford ML, Tomlinson LA, Weaving G, Rocks BF, Rajkumar C and Holt SG. Instability of fibroblast growth factor-23 (FGF-23): implications for clinical studies. *Clin Chim Acta*. 2011;412:1008-11.

3. White KE, Carn G, Lorenz-Depiereux B, Benet-Pages A, Strom TM and Econs MJ. Autosomal-dominant hypophosphatemic rickets (ADHR) mutations stabilize FGF-23. *Kidney Int*. 2001;60:2079-86.
4. Faul C, Amaral AP, Oskouei B, Hu MC, Sloan A, Isakova T, Gutierrez OM, Aguillon-Prada R, Lincoln J, Hare JM, Mundel P, Morales A, Scialla J, Fischer M, Soliman EZ, Chen J, Go AS, Rosas SE, Nessel L, Townsend RR, Feldman HI, St John Sutton M, Ojo A, Gadegbeku C, Di Marco GS, Reuter S, Kentrup D, Tiemann K, Brand M, Hill JA, Moe OW, Kuro OM, Kusek JW, Keane MG and Wolf M. FGF23 induces left ventricular hypertrophy. *J Clin Invest*. 2011;121:4393-408.
5. de Jong MA, Mirkovic K, Mencke R, Hoenderop JG, Bindels RJ, Vervloet MG, Hillebrands JL, van den Born J, Navis G, de Borst MH and consortium N. Fibroblast growth factor 23 modifies the pharmacological effects of angiotensin receptor blockade in experimental renal fibrosis. *Nephrol Dial Transplant*. 2016.
6. Hewitson TD and Grimwood L. Immuno and lectin histochemistry for renal light microscopy. *Methods Mol Biol*. 2009;466:133-47.
7. Yamazaki M, Kawai M, Miyagawa K, Ohata Y, Tachikawa K, Kinoshita S, Nishino J, Ozono K and Michigami T. Interleukin-1-induced acute bone resorption facilitates the secretion of fibroblast growth factor 23 into the circulation. *J Bone Miner Metab*. 2015;33:342-54.
8. Fang Y, Ginsberg C, Seifert M, Agapova O, Sugatani T, Register TC, Freedman BI, Monier-Faugere MC, Malluche H and Hruska KA. CKD-induced wingless/integration1 inhibitors and phosphorus cause the CKD-mineral and bone disorder. *J Am Soc Nephrol*. 2014;25:1760-73.
9. Spichtig D, Zhang H, Mohebbi N, Pavik I, Petzold K, Stange G, Saleh L, Edenhofer I, Segerer S, Biber J, Jaeger P, Serra AL and Wagner CA. Renal expression of FGF23 and peripheral resistance to elevated FGF23 in rodent models of polycystic kidney disease. *Kidney Int*. 2014;85:1340-50.
10. Hewitson TD, Christie M and Smith ER. Laser Capture Microdissection of Archival Kidney Tissue for qRT-PCR. *Methods Mol Biol*. 2016;1397:251-265.
11. Fuller JA, Brun-Zinkernagel AM, Clark AF and Wordinger RJ. Subtilisin-like proprotein convertase expression, localization, and activity in the human retina and optic nerve head. *Invest Ophthalmol Vis Sci*. 2009;50:5759-68.
12. Grimwood L and Masterson R. Propagation and culture of renal fibroblasts. *Methods Mol Biol*. 2009;466:25-37.
13. Hayashi H, Abdollah S, Qiu Y, Cai J, Xu YY, Grinnell BW, Richardson MA, Topper JN, Gimbrone MA, Jr., Wrana JL and Falb D. The MAD-related protein Smad7 associates with the TGFbeta receptor and functions as an antagonist of TGFbeta signaling. *Cell*. 1997;89:1165-73.
14. Arking DE, Krebsova A, Macek M, Sr., Macek M, Jr., Arking A, Mian IS, Fried L, Hamosh A, Dey S, McIntosh I and Dietz HC. Association of human aging with a functional variant of klotho. *Proc Natl Acad Sci U S A*. 2002;99:856-61.
15. Hewitson TD, Boon WC, Simpson ER, Smith ER and Samuel CS. Estrogens do not protect, but androgens exacerbate, collagen accumulation in the female mouse kidney after ureteric obstruction. *Life Sci*. 2016;158:130-6.
16. Giordano A, Romano S, Mallardo M, D'Angelillo A, Cali G, Corcione N, Ferraro P and Romano MF. FK506 can activate transforming growth factor-beta signalling in vascular smooth muscle cells and promote proliferation. *Cardiovasc Res*. 2008;79:519-26.
17. Touchberry CD, Green TM, Tchikrizov V, Mannix JE, Mao TF, Carney BW, Girgis M, Vincent RJ, Wetmore LA, Dawn B, Bonewald LF, Stubbs JR and Wacker MJ. FGF23 is a

novel regulator of intracellular calcium and cardiac contractility in addition to cardiac hypertrophy. *Am J Physiol Endocrinol Metab*. 2013;304:E863-73.
18. Zhu J-N, Chen R, Fu Y-H, Lin Q-X, Huang S, Guo L-L, Zhang M-Z, Deng C-Y, Zou X, Zhong S-L, Yang M, Zhuang J, Yu X-Y and Shan Z-X. Smad3 Inactivation and MiR-29b Upregulation Mediate the Effect of Carvedilol on Attenuating the Acute Myocardium Infarction-Induced Myocardial Fibrosis in Rat. *PLoS ONE*. 2013;8:e75557.

## Supplementary Figures

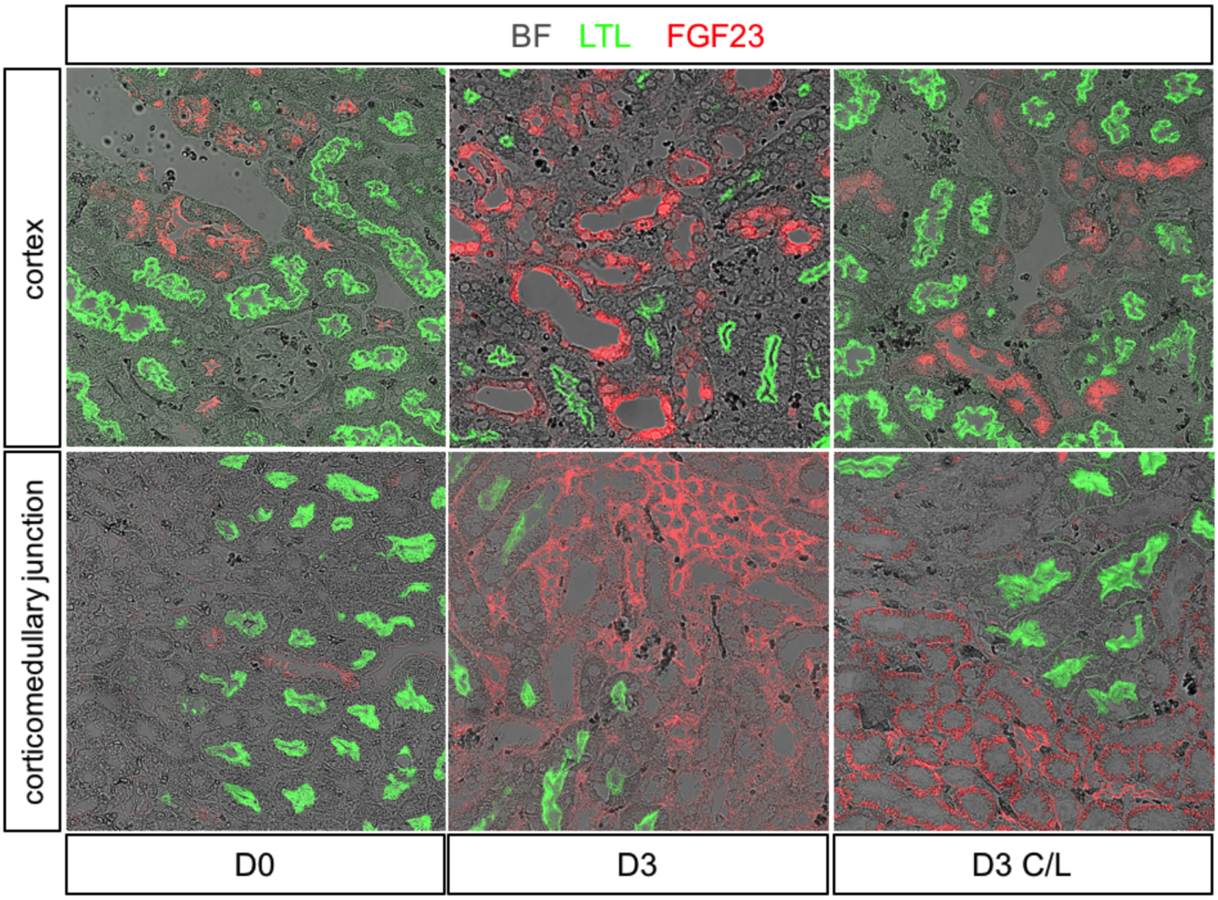


**Figure S1: Renal localisation of FGF23 before and after UUO.**

A comparison of immunofluorescent FGF23 staining using brightfield (BF) confocal microscopy of outer cortex (upper panel) and corticomedullary junction (lower panel) in in D0, D3 OB and D3 C/L kidney sections. Scale bar=50μm.

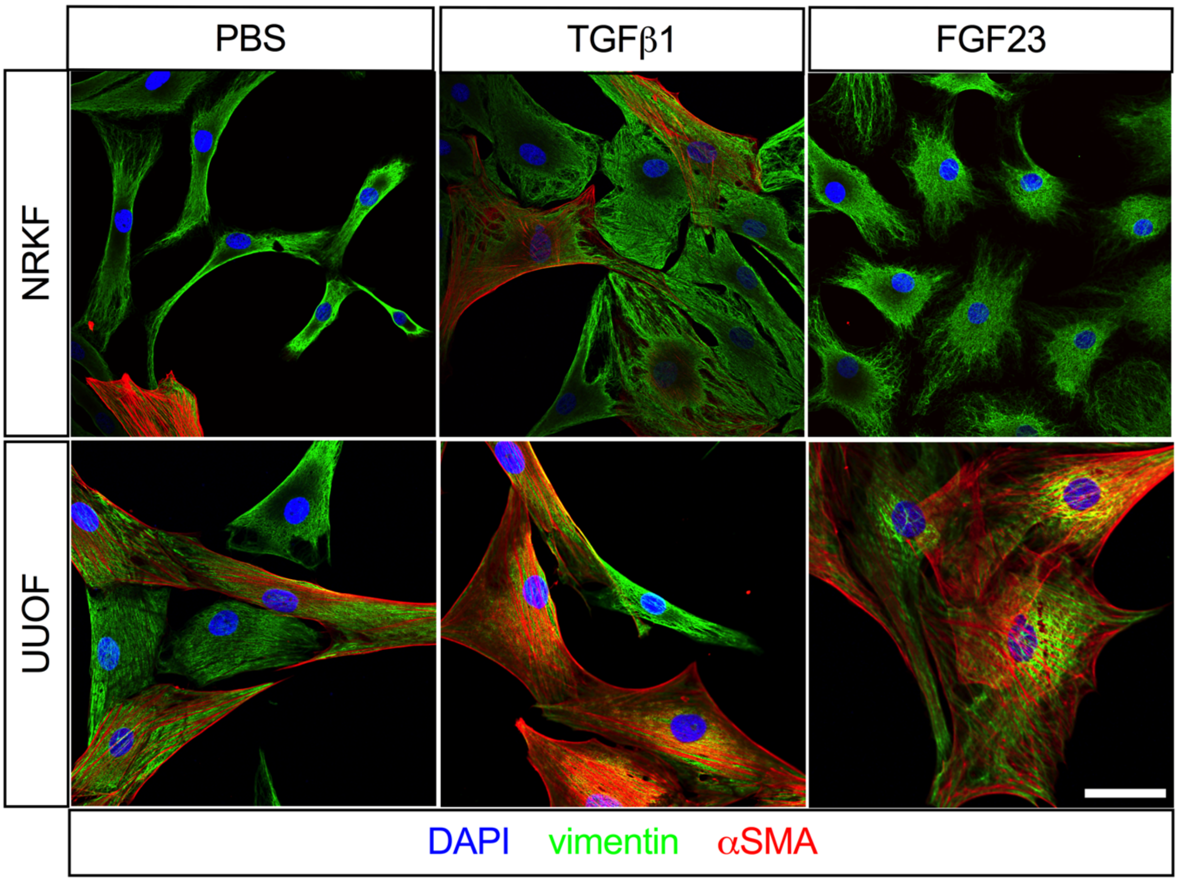


**Figure S2: FGF23 stimulates renal fibroblast activation.**

Representative immunofluorescent staining of cytoskeletal proteins vimentin (green) and αSMA (red) in fibroblasts cultured from D0 control (NRKF) and D3 OB (UUOF) rat kidney explants treated for 48h with either TGFβ1 or FGF23 (both at 1 ng/mL). Nuclei were stained with DAPI (blue). Scale bar =50μm.

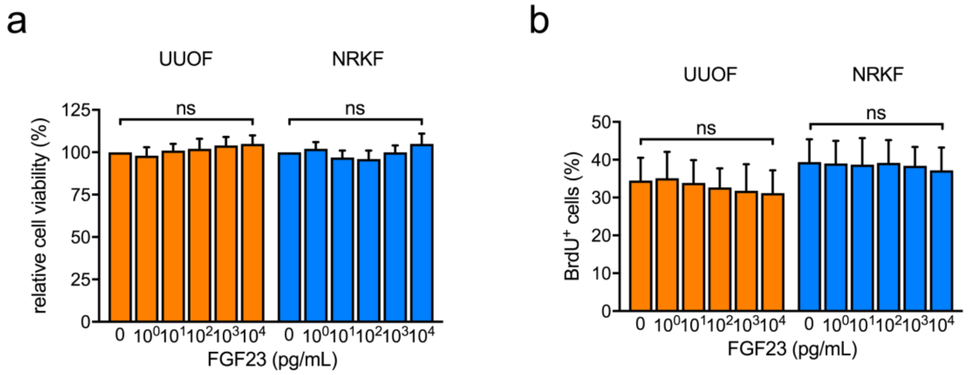


**Figure S3: FGF23 has no effect on viability of proliferation in UUOF or NRKF.**

**(a)** Flow cytometric analysis of relative cell viability (TO-PRO-3 exclusion) and apoptosis (AnnexinV binding) in both cell types following 48hr treatment with 10 pg/ml- 10 ng/ml FGF23. Results are expressed relative to vehicle treated cells. **(b)** Flow cytometric analysis of changes in proliferation after treatment with FGF23 (10pg/ml – 10 ng/ml), relative to vehicle. Proliferation was assessed from % bromodeoxyuridine (Brdu) incorporation over 48 hrs. Data are shown as mean ± SD and represent at least two independent experiments with similar results. ns, not significant. P-values were determined by one-way ANOVA with Holm-Sidak multiple comparisons test.

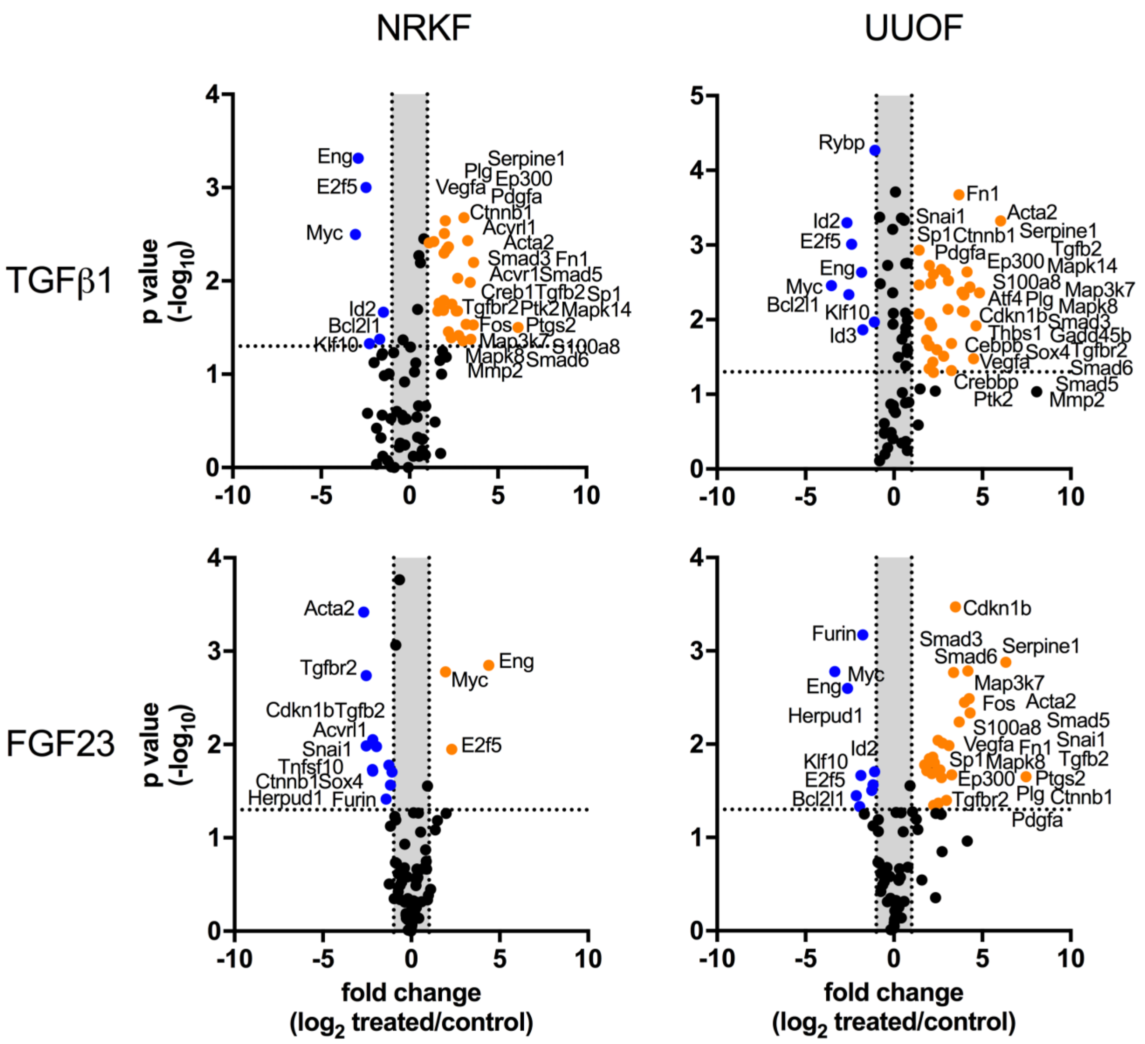


**Figure S4: Divergent effects of FGF23 on TGFβ1 signalling in NRKF and UUOF.**

Volcano plots showing qRT-PCR profiling of TGFβ1 signalling target gene expression in NRKF and UUOF treated for 24h with either TGFβ1 or FGF23 (both at 1 ng/mL for 24h) or vehicle (PBS). Transcripts showing greater than 2-fold change in expression compared to vehicle at a P-level <0.05 were considered significant and labelled (down-regulated, blue; up-regulated, orange). Results are representative of two independent experiments.

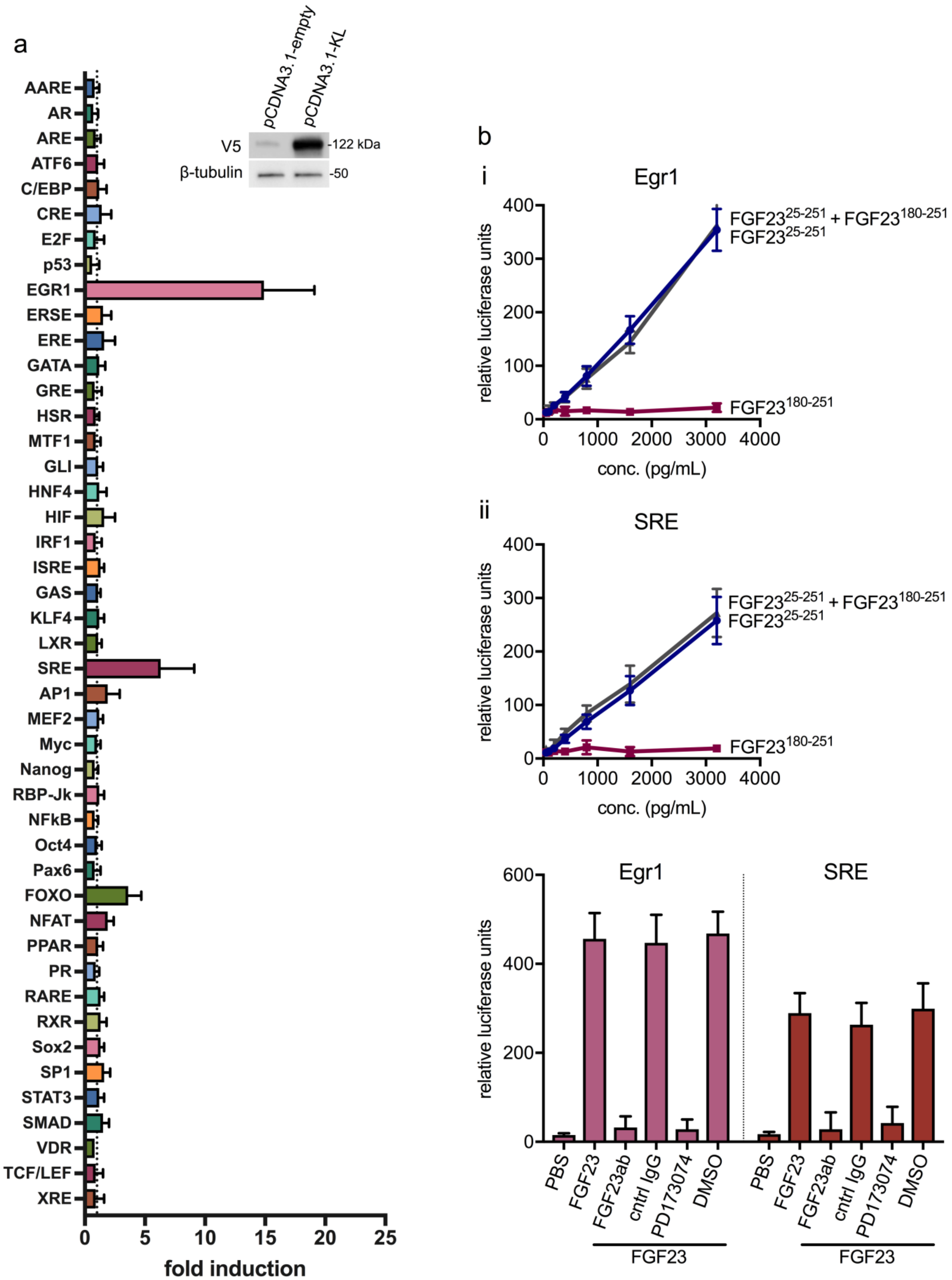


**Figure S5: Validation of FGF23 bioactivity in αKl expressing HEK293 cells.**

**(a)** FGF23 induced changes in 45 signal transduction pathways in HEK293 cells transiently expressing V5-tagged membrane αKl. Cells were treated for 1 ng/ml FGF23 for 30 min, with results expressed relative to vehicle treatment. Western blot analysis was used to confirm CMV overexpression of αKl (upper right). **(b)** Changes in activation of Egr1 (i) and SRE (ii) pathways with increasing doses of full length intact FGF23, the isolated c terminal fragment, or in combination with the c-terminal fragment in 10 molar excess. **(c)** Inhibition of FGF23 induced (1 ng/ml for 30 min) activation of Egr1 and SRE by pre-treatment with PD1730374, or treatment of cells with FGF23 pre-absorbed with a 100-fold molar excess of FGF23 blocking antibody (#AF2604; R&D Systems) vs vehicle (DMSO) or control IgG respectively.

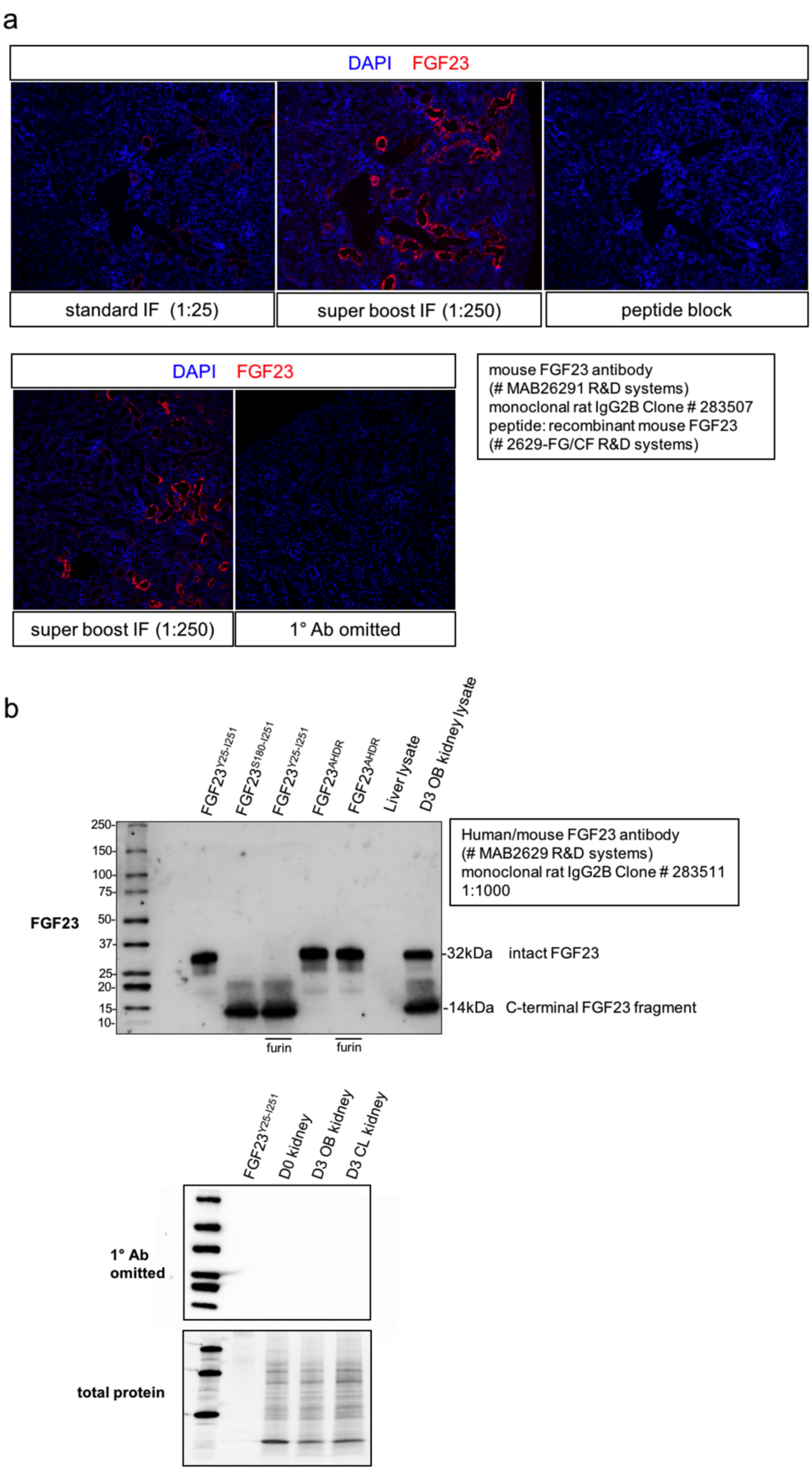


**Figure S6: Validation of FGF23 antibodies used for immunofluorescence and western blotting.**

**(a)** Cortical serial sections of kidney tissue (D3 OB) incubated with anti-FGF23 (red) (i), anti-FGF23 with Superboost amplification (ii), and anti-FGF23 pre-absorbed with a 10-molar excess of immunising peptide (iii). A second technical control where the primary antibody has been omitted is also shown. Nuclei stained with DAPI (blue). Scale bar=100μm**.**

**(b)** Western blotting of recombinant wild-type intact (~32kDa), C-terminal FGF23 (~14 kDa), mutant non-cleavable ADHR FGF23 (~32kDa) with or without pre-treatment with furin (Sigma). Whole tissue lysates of liver (negative control) and D3 OB kidney were run alongside. Peptides were prepared in 1% BSA as a carrier. Membranes were probed with monoclonal rat ant-mouse FGF23 antibody (#MAB2629). Blot underneath shows the absence of non-specific staining of peptide and kidney tissue lysates with the primary antibody omitted. Total protein stain-free blot is used to demonstrate loading.

## Supplementary Tables

### Table S1 - Inhibitors

| Inhibitor | Concentration (reference) | Cat # | Supplier |
|---|---|---|---|
| PD173074 | 10 nM [4] | P2499 | Sigma-Aldrich, St. Louis, MO, USA |
| SB431542 | 5 nM [16] | 1614 | Tocris, Bristol, UK |
| PD166866 | 50 nM [17] | PZ0114 | Sigma-Aldrich, St. Louis, MO, USA |
| Naringenin | 80 µM [18] | 14173 | Cayman Chemical, Ann Arbor, MI |

None of the compounds were found to significantly reduce cell viability (MTT assay) over 24hr, when used at the stated concentrations.

**Table S2 -Primary antibodies**

Antibodies used at the stated dilutions for immunochemistry (IHC), immunofluorescence (IF), flow cytometry (FC) or Western blotting (WB)

| **Antibody** | **Application/ dilution** | **Cat ##** | **Supplier** |
|---|---|---|---|
| rabbit polyclonal anti-β-tubulin | WB 1:2000 | ab6046 | Abcam, Cambridge, UK), |
| mouse monoclonal anti-BrdU PerCP-Cy™5.5 | FC 1:20 | | BD Biosciences, San Diego, USA |
| rabbit polyclonal anti-mouse collagen I | IHC 1:500 | T40777R | Biodesign, Saco, ME, USA |
| rat monoclonal anti-mouse FGF23 | IF 1:300 | MAB26291 clone 283507, | R&D systems |
| rat monoclonal anti-mouse FGF23 | WB 1:1000 | MAB2629, clone 283511 | R&D Systems |
| goat polyclonal anti-human/mouse FGF23 | B/N 1:10 | AF2604 | R&D Systems |
| rabbit monoclonal anti-FGFR1 | WB 1:1000; FC 1:100 | 4740 clone D8E4 | Cell Signalling Technology |
| mouse monoclonal anti-FGFR2 | FC 1:50 | ab58201 | Abcam |
| rabbit polyclonal anti-FGFR2 | WB 1:400 | Sc-122 C17 | Santa Cruz Biotechnology, Santa Cruz, CA, USA |
| mouse monoclonal anti-FGFR3 | FC 1:50; | FAB766P | R&D Systems |
| rabbit polyclonal anti-FGFR3 | WB 1:500; | Sc-123 | Santa Cruz Biotechnology |
| mouse monoclonal anti-FGFR4 | FC 1:50 | ab44971 | Abcam |
| mouse monoclonal anti-FGFR4 | WB 1:500 | Sc-136988 | Santa Cruz Biotechnology |
| mouse monoclonal anti-HA | WB 1:10000 | H3663 clone HA-7, | Sigma |
| rat monoclonal anti-αKL | WB 1:500 | KO603 | Transgenic Inc, Kobe, Japan |
| goat polyclonal anti-βKL | WB 1:500 | | R&D Systems |
| rabbit monoclonal anti-pSmad2(Ser465/467)/pSmad3 (Ser423/425) PE | FC 1:400 | 11979 | Cell Signalling Technology |
| mouse anti-α-smooth muscle actin | IHC 1:200; IF 1:250 | MO851 | Dako, Glostrup, Denmark |
| rabbit monoclonal anti-α- | FC 1:50 | 202296 | Abcam |

| | | | |
|---|---|---|---|
| smooth muscle actin Alexa Fluor 647 | | | |
| rabbit polyclonal anti-TGFBR1 | FC 1:25 | 31013 | Abcam |
| mouse monoclonal anti-TGFBR2 | FC 1:50 | ab78419 | Abcam |
| mouse monoclonal anti-V5 | WB 1:10000 | H8012 clone V5-10 | Sigma |
| rabbit monoclonal anti-vimentin | IHC 1:500, IF 1:250 | ab92547 | Abcam |

**Table S3 -Secondary antibodies**

| Antibody | Application/ dilution | Cat # | Supplier |
|---|---|---|---|
| goat anti-mouse IgG H&L AlexaFluor 594 | IF: 1:500 | A-11032 | Life Technologies |
| goat anti-rabbit IgG H&L AlexaFluor 488 | IF: 1:500 | A-11008 | Life Technologies |
| goat anti-mouse IgG H&L Dylight 488 preabsorbed | FC 1:500 | ab96879 | Abcam |
| goat anti-rabbit IgG H&L AlexaFluor 488 preabsorbed | FC 1:1000 | ab150077 | Abcam |
| goat anti-rabbit IgG H&L (HRP) preadsorbed | WB 1:10000 | ab97051 | Abcam |
| goat anti-mouse IgG H&L (HRP) preadsorbed | WB 1:10000 | ab97023 | Abcam |

**Table S4 – primers**

**1a Mouse qRT-PCR primers**

| **Target** | **Forward (5' to 3')** | **Reverse (5' to 3')** |
|---|---|---|
| *Col1a1* | GGCACAGACGGCTGAGTAGG | TCTGACTGGAAGAGCGGAGAG |
| *Col4a1* | GCCTTCCGGGCTCCTCAG | TTATCACCAGTGGGTCCG |
| *Tgfb1* | GCTCGCTTTGTACAACAGCACC | GCGGTCCACCATTAGCACG |
| *Acta2* | TCCAGAGTCCAGCACAATACCAGT | TGACAGAGGCACCACTGAACC |
| *Fn1* | TGCAAGGCAACCACACTGAC | GATGGAATCCGGGAGCTTTT |
| *Fgfr1* | TGTTTGACCGGATCTACACACA | CTCCCACAAGAGCACTCCAA |
| *Fgfr2* | TCGCATTGGAGGCTATAAGG | CGGGACCACACTTTCCATAA |
| *Fgfr3* | GCATCCTCACTGTGACATCAAC | CCTGGCGAGTACTGCTCAAA |
| *Fgfr4* | CGCCAGCCTGTCACTATACAAA | CCAGAGGACCTCGACTCCAA |
| *Kl* | AGCGATAGTTACAACAAC | GCATTCTCTGATATTATAGTC |
| *Npt2a* | ATGCTGGCTTTCCTTTAC | CCACAATGTTCATGCCTTCT |
| *Npt2c* | CGTGCGGACTGTTATCAATG | TACTGGGCAGTCAGGTTTCC |
| *Cyp27b1* | ACACTTCGCACAGTTTACG | TTAGCAATCCGCAAGCAC |
| *Cyp24a1* | GTTCTGTCCACGGTAGGC | CCAGTCTTCGCAGTTGTCC |
| *furin* | CATGACTACTCTGCTGATGG | GAACGAGAGTGAACTTGGTC |
| *Pc2* | CGAGAGTTTCCGTAGGGTGG | CATCAGAACAGCAGGCTGGG |
| *Pc7* | TCCTCGATGACGGCATAGAAA | TTCTCGTTGCTGGCGTCATAT |

| | **Bio-Rad PCR Prime Unique assay ID** |
|---|---|
| *Fgf23* | qMmuCID0008822 |
| *Actb* | qMmuCED0027505 |

**1b. Rat qRT-PCR primers**

| | **Bio-Rad PCR Prime Unique assay ID** | |
|---|---|---|
| *Col1a1* | qRnoCED0007857 | |
| *Col4a1* | qRnoCED0003647 | |
| *Tgfb* | qRnoCID0009191 | |
| *Fn1* | qRnoCID0007136 | |
| *Actb* | qRnoCID0056984 | |
| | | |
| | **Forward (5' to 3')** | **Reverse (5' to 3')** |
| *Acta2* | AGATCACAGCCCTCGCT | AGAATATTTGCGTTCTGGAG |
